# miR-211 facilitates platinum chemosensitivity by blocking the DNA damage response (DDR) in ovarian cancer

**DOI:** 10.1038/s41419-019-1715-x

**Published:** 2019-06-24

**Authors:** Tianzhen Wang, Dapeng Hao, Shucai Yang, Jianhui Ma, Weiwei Yang, Yuanyuan Zhu, Mingjiao Weng, Xiang An, Xuefei Wang, Yafei Li, Di Wu, Jing Tang, Chao Yang, Yan He, Lei Zhang, Xiaoming Jin, Guangyu Wang, Zhiwei Li, Tongsen Zheng, Hongxue Meng, Yukuan Feng, Xiaobo Li

**Affiliations:** 1 Department of Pathology, Harbin Medical University, Harbin, 150081 China; 2 Faculty of Health Sciences, University of Macau, Macau, China; 3 Department of Anatomy, Harbin Medical University, Harbin, 150081 China; 4 Department of Obstetrics and Gynecology, First Affiliated Hospital of Harbin Medical University, Harbin, 150001 China; 5 Department of Gastrointestinal Medical Oncology, Harbin Medical University Cancer Hospital, Harbin, 150081 China; 6 Department of Pathology, Harbin Medical University & Harbin Medical University Cancer Hospital, Harbin, 150081 China; 7 Key Laboratory of Heilongjiang Province for Cancer Prevention and Control, School of Basic Medicine, Mudanjiang Medical University, Mudanjiang, 157011 China; 8 North China Translational Medicine Research and Cooperation Center (NTMRC), Harbin, 150081 China

**Keywords:** Chemotherapy, Colon cancer

## Abstract

The DNA damage response (DDR) is one of the most important mechanisms of platinum resistance in ovarian cancer. Some miRNAs have been identified to be involved in the regulatory network of DDR, thus the abnormal expression of miRNAs might affect platinum chemosensitivity in ovarian cancer. In this study, by assessing miRNAs simultaneously targeting a set of DDR genes that exhibited response to platinum, we found that miR-211 inhibited most of those genes, and proposed that miR-211 might affect the sensitivity of ovarian cancer cells to platinum by targeting multiple DDR genes and thereby determine the prognosis of ovarian cancer. To verify the hypothesis, we analyzed the association between miR-211 level and clinical prognosis, assessed the effect of miR-211 on DDR and platinum chemosensitivity, and explored the possible molecular mechanism. We revealed that miR-211 enhanced platinum chemosensitivity and was positively correlated with favorable outcomes in ovarian cancer patients. Many DDR genes including TDP1 were identified as targets of miR-211. In contrast, TDP1 suppressed DNA damage and platinum chemosensitivity. Moreover, the miR-211 level in tissues was shown to be associated with the good outcome of neoadjuvant chemotherapy and negatively correlated with the expression of TDP1. Conclusively, we demonstrated that miR-211 improves the prognosis of ovarian cancer patients by enhancing the chemosensitivity of cancer cells to platinum via inhibiting DDR gene expression, which provides an essential basis to identify novel treatment targets to block DDR effectively and improve chemosensitivity in ovarian cancer.

## Introduction

Ovarian cancer is the leading cause of cancer death among gynecological malignancies^1^. It was estimated that 22,240 cases of ovarian cancer would be newly diagnosed, and that 14,070 affected patients would die in America in 2018^2^. The standard treatment for ovarian cancer is surgical operation followed by multiple cycles of platinum-based chemotherapy. Platinum agents induce DNA crosslinks and block DNA duplication and transcription in proliferating cells^3,4^. The application of platinum has effectively improved the prognosis of ovarian cancer. However, some ovarian cancer patients exhibit no response to platinum, and most patients will relapse in a few years with developed chemoresistance^5,6^.

In recent years, many investigators have devoted themselves to the mechanism of chemoresistance. Numerous studies have shown that the enforced DNA damage response (DDR) is one of the most important mechanisms for resistance to chemotherapy-based platinum agents^7,8^. Patients with DDR deficiency exhibit a positive response to chemosensitivity. For example, patients with BRCA1/2 mutations are more sensitive to platinum treatment^9,10^. Targeting DDR genes in combination with DNA-damaging chemotherapeutic drugs is a promising treatment for cancer^11^.

MicroRNA (miRNA) is a type of short noncoding RNA that is 20~25 nucleotides in length and can induce target mRNA degradation or translation suppression by binding to complementary sequences in the 3′-untranslated region (3′-UTR) of mRNA^12,13^. The abnormal expression of miRNAs is involved in the regulatory network of DDR^14,15^. For example, miR-421 inhibits ATM expression, and miR-146 downregulates BRCA1^16,17^. More importantly, it is common that one miRNA regulates the expression of multiple genes simultaneously by the above mechanism. Can some miRNAs target a set of DDR genes and thereby become the key point of treatment to inhibit DDR and improve chemosensitivity? We assessed the inhibitory ability of miRNAs on DDR genes that exhibited response to platinum in previous studies and ranked these miRNAs according to the number of DDR genes inhibited. We found that miR-211 inhibited the most genes. Based on these findings, we hypothesized that miR-211 might affect the sensitivity of ovarian cancer cells to platinum by targeting multiple DDR genes and thereby determine the prognosis of ovarian cancer. To verify this hypothesis, we analyzed the association between miR-211 level and clinical prognosis, assessed the effect of miR-211 on DDR and platinum chemosensitivity, and explored the possible molecular mechanism. This study provides an essential basis for identifying novel treatment targets to block DDR effectively and improve chemosensitivity in ovarian cancer.

## Materials and methods

### Datasets and data processing

The TCGA data of ovarian cancer samples were downloaded from the GDSC Broad Institute (http://firebrowse.org; downloaded date: 7/9/2014). The TCGA cohort, including 594 ovarian cancer samples with miRNA expression data, was used to analyze the prognostic value of miR-211. The targets of miR-211 were predicted using the miRTarBase (http://mirtarbase.mbc.nctu.edu.tw/). The targets of miR-211 that are negatively correlated with the expression of miR-211 were extracted from 261 TCGA ovarian cancer samples with both mRNA and miRNA expression data. We utilized data from 14 studies, which explored the association between mRNA expression and ovarian cancer overall survival (OS) to assess the effect of the miR-211 targets on OS. Consequently, this information could indirectly reflect the correlation between miR-211 and prognosis in ovarian cancer. In view of the function of miR-211 targets, 319 samples with aggregated and normalized mutation data were used to identify the correlation of miR-211 with genome instability.

### Human tissues

A total of 30 formalin-fixed paraffin-embedded high grade serous ovarian cancer samples were collected from the Pathology Department of Harbin Medical University Cancer Hospital from March to July 2017. The diagnosis was confirmed by at least two pathologists. All patients received neoadjuvant chemotherapy based on carboplatin before surgery. The therapy responses were evaluated according to computed tomography, which indicated a significant reduction in tumor volume using Response Evaluation Criteria In Solid Tumors (RECIST)^18^, or by a histopathological analysis, which assessed the presence of residual tumor cells and the extent of fibrosis in resected tumor tissues^19^. Thus, 16 of these cases were responders, whereas the other 14 cases were non-responders. The clinical information of the patients is presented in Supplementary Table 1. Five-micrometer-thick serial sections were cut from these samples to detect the expression of miR-211 and TDP1. All patients signed informed consent prior to their inclusion in the study. The Ethical Committee of Harbin Medical University approved this study.

### Immunohistochemistry

TDP1 expression was detected by immunohistochemistry. Briefly, the sections were deparaffinized in xylene, rehydrated in a series of graded alcohol and rinsed in distilled water. Endogenous peroxidase in tissues was blocked by 3% H_2_O_2_ at room temperature (RT) for 10 min. Antigen retrieval was performed by immersing tissue sections in phosphate buffer saline (PBS, pH 7.4) in a microwave oven. Nonspecific reactions were blocked by 5% bovine serum albumin (BSA) at RT for 20 min. Then, the sections were incubated with primary mouse monoclonal antibody against TDP1 protein (Santa Cruz Biotechnology, CA, USA. No. sc-365674, diluted by 1:200) at 4 °C overnight and linked with goat anti-mouse secondary antibody (ZSGB Bio, Beijing, China) at 37 °C for 1 h. Developing chromogen with 3,3′-diaminobenzidine (DAB) (ZSGB Bio, Beijing, China) was used to mark their combination. TDP1 was mainly expressed in the nucleus, and a positive result was noted when >5% of cells were stained. The result was evaluated based on the area and intensity of the stain as previously described by two pathologists who were blinded to the clinical information^20^. A negative control was created by replacing the primary antibodies with PBS.

### In situ hybridization

The level of miR-211 expression in ovarian cancer tissues was determined with an in situ hybridization kit (Bioster Biological Technology Co., Ltd, Wuhan, China: No. MK10501). First, these sections were deparaffinized and rehydrated. Then, 3% H_2_O_2_ was used to inactivate endogenous peroxidase. Then, miRNA was exposed to proteinase-K at 37 °C for 15 min. The sections were incubated with preliminary hybrid liquid and then hybridized with a DIG-labeled miR-211 antisense probe (Supplementary Table 2) at 42 °C overnight. After washing and blocking, the sections were incubated with anti-DIG antibody at 37 °C for 60 min. SABC and biotin peroxidase were used to mediate DAB staining according to the manufacturer’s protocol. Positive staining was noted in the cytoplasm. The result was scored by the same methods that described for immunohistochemistry. The means were used for statistical analysis. The patients were separated into two groups (high level and low level) based on the median miR-211 level.

### Cell culture

Two ovarian cancer cell lines SKOV3^21^ and HO8910^22^ were used in this study to investigate the effect of miR-211 on the chemosensitivity of ovarian cancer to carboplatin. In addition, 293TN cells were used in the Dual-Luciferase Reporter Assay. All cell lines were cultured using Dulbecco’s modified Eagle medium (DMEM; HyClone, Logan, UT, USA) with 10% fetal bovine serum (FBS; Invitrogen, Carlsbad, CA, USA), 100 μg/ml streptomycin, and 100 IU/ml penicillin at 37 °C in a humidified atmosphere containing 5% CO_2_.

### Reagent and transfection

The miR-211 mimic, miR-211 inhibitor and negative control (NC) were synthesized and purchased from GenePharma (Suzhou, Zhejiang, China). The sequences are presented in Supplementary Table 2. All these molecules were transfected into cells using Lipofectamine 2000 (Invitrogen) according to the manufacturer’s protocol.

### Cell viability assay

Eight hours after transfection, the cells were seeded into 96-well plates and then treated with gradient carboplatin (Qilu Pharmaceutical Co., Jinan, Shandong, China) for 48 h. Cellular viability was assessed using a luciferase-based ATP quantitation assay (CellTiter-Glo™, Promega, Madison, WI, USA) in quadruplicate as previously described^23^. In brief, the plate was equilibrated at RT for 30 min. Then, 100 μl CellTiter-Glo reagent was added into each well, and the contents were mixed on an orbital shaker. Finally, the plate was incubated at RT for 10 min in a darkroom, and the value of luminescence was detected on a SpectraMax^®^ M5 Multi-Mode Microplate Reader. The blank control was used to normalize the value. All assays were replicated three times.

### Tumor xenograft studies

Ovarian cancer cells (1.5 × 10^6^ cells resuspended in 100 μl PBS per side) were subcutaneously inoculated into both flanks of 5- to 6-week-old female nude mice (Vital River Laboratory, Beijing, China), and each cell line was inoculated 10 mice. At the 6th day after inoculation when the average tumor size was around 6 mm^3^, the mice underwent agomiR (RiboBio, Guangdong, China) treatment. The agomiR-211(right) and agomiR-NC (left) were injected intratumorally at a dose of 1 nmol diluted in 15 μl PBS per tumor every 4 days seven times. These mice were randomly divided into two groups, five mice in one group were synchronously administrated PBS, whereas five mice in the other group were administrated 25 mg/kg carboplatin intraperitoneally every 4 days for seven rounds, respectively. Finally, the mice were sacrificed by cervical dislocation under anesthesia. Tumor volumes were calculated as (length × width × width)/2. All animal experiments were performed in accordance with the Guide for the Care and Use of Laboratory Animals of Harbin Medical University.

### Plasmid construction and dual-luciferase reporter assay

The 3′-UTR of the target gene, which contains the binding sites of miR-211, was amplified by PCR from genomic DNA. The primers are presented in Supplementary Table 2. The sequence was inserted into the pGL3-LUC reporter vector (Promega). Briefly, 293TN cells were seeded into 96-well plates at a density of 2 × 10^4^ cells per well. Twenty-four hours later, the cells were cotransfected with pGL3-LUC reporter vectors, pRL-TK Renilla luciferase reporter vectors (Promega) and miR-211 mimics or NC. Transfection was performed using Lipofectamine 2000 (Invitrogen). Forty-eight hours posttransfection, Firefly and Renilla luciferase were detected using Promega GloMax® 20/20 Luminometer E5311. All assays were replicated three times.

### Construction of stable cell lines by lentiviral infection

shRNA against TDP1 was designed and synthesized according to the known sequence and then inserted into lentiviral expression vector. These vectors were transfected into 293TN cells together with packing vectors to obtain pseudo lentiviral particles. SKOV3 and HO8910 cells were incubated with 10 MOI of GFP lentiviral particles for at least 12 h. After conventional culture for 72 h, the cells were sorted by flow cytometry. The sequence of shRNA targeting TDP1 is presented in Supplementary Table 2.

### Comet assay

Comet assay was used to detect DNA damage. In brief, the slides were coated with agarose by dipping them into 0.75% melted agarose solution. Cells were resuspended in PBS (1 × 10^6^/ml), and 10 μl of cell suspension was added to 90 μl low melting point agarose. After gently mixing, the mixture was placed on the slide. After solidification, the slide was placed in lysis solution for 1 h at 4 °C. The slide was washed with electrophoresis buffer and electrophoresis was performed at 50 V for 5 min. After washing, cells were fixed with absolute ethanol, and the slide was dried at 37 °C for 10 min. The slide was incubated with Gel stain for 10 min in a darkroom. After washing the slide, the fluorescence of DNA was recorded by fluorescence microscope (Nikon Eclipse E800, Japan) with digital camera (Nikon DXM1200F, Japan). The DNA damage degree was evaluated based on the percentage of DNA in the comet tail, which was calculated according to the ratio of fluorescence intensity of the comet tail to the whole comet. At least five DNA comets were randomly selected and calculated to indicate the DNA damage degree induced by carboplatin^24^. All assays were replicated three times. The differences of comet assay between groups were analyzed by unpaired T-test.

### Western blot analysis

Total proteins were extracted from ovarian cancer cells using RIPA buffer (Beyotime Institute of Biotechnology, Shanghai, China). Proteins were quantified using a BCA Kit (Beyotime Institute of Biotechnology). Equal protein was separated on an SDS-PAGE gel by electrophoresis and transferred onto a PVDF membrane. Then, 5% BSA was used to block the blots at 4 °C overnight. Then, membranes were incubated with the following primary antibodies: TDP1 mouse monoclonal antibody diluted at 1:500 (Santa Cruz Biotechnology, CA, USA: No. sc-365674), γ-H2AX (phospho-histone H2AX at ser139), rabbit polyclonal antibody diluted at 1:500 (Affinity Bioscience, Changzhou, China: No. AF3187), β-actin mouse monoclonal antibody diluted at 1:5000 (ZSGB Bio, Beijing, China: No. TA-09), and GAPDH mouse monoclonal antibody diluted at 1:5000 (Proteintech, IL, USA: No. 60004–1–1g). After washing, the membranes were incubated with peroxidase conjugated secondary antibody (Santa Cruz Biotechnology) for 1 h at 37 °C. The ECL system (ThermoScientific, Rockford, IL, USA) was used to visualize the blots. To detect γ-H2AX, we added phosphatase inhibitors to RIPA before protein collection. All assays were replicated three times.

### Statistical analysis

The miR-211 median obtained from in situ hybridization served as the threshold value for grouping. The variables between two groups (miR-211 level, TDP1 level, and dual-luciferase reporter assay) were analyzed using a two-sided Student’s *t* test. The association between miR-211 and the response to platinum was analyzed by Fisher’s exact test. The tumor size and chemosensitivity were analyzed using analysis of variance. The difference in the mutation frequency between the two groups was evaluated using the Mann-Whitney U test. Survival analysis were conducted with the Kaplan-Meier method using the log-rank test, and the best separation based on the expression of miR-211 is presented. Univariate and multivariate analyses with Cox regression were used to determine the proportional hazards. The DAVID Bioinformatics Tool (https://david.ncifcrf.gov/, version 6.7) was used to complete the functional enrichment analysis of the gene ontology (GO). All statistical analysis were performed using SSPS 17.0. *p* < 0.05 was considered statistically significant.

## Results

### miR-211 is positively correlated with response to platinum and ovarian cancer prognosis

**Fig. 1:**
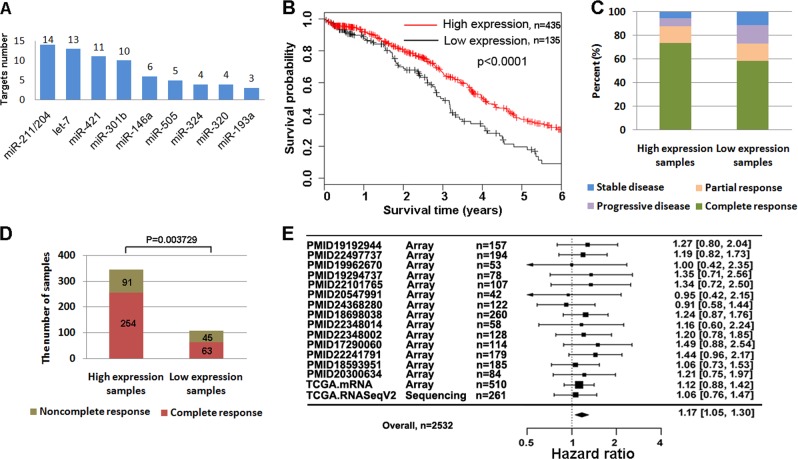
miR-211 was positively correlated with the response to platinum and prognosis of ovarian cancer. **a** In total, 14 out of 80 DDR genes exhibiting response to platinum were targeted by miR-211 according to miRTarBase. **b** Patients with high miR-211 levels exhibited significantly longer survival time than those with low miR-211 levels. **c** miR-211 improved the response of ovarian cancer patients to platinum. **d** More patients exhibited acomplete response to platinum in the group with high miR-211 levels than in the group with low miR-211 levels. **e** Patients with high expression of miR-211 target genes exhibited worse survival in most datasets

We collected 80 DDR genes that exhibited a response to platinum drugs in previous studies (Supplementary Table 3) and investigated miRNAs involved in regulating these DDR genes^25,26^. Then we ranked miRNAs according to the number of DDR genes simultaneously targeted by them, and found that miR-211/miR-204, let-7, miR-421 may simultaneously target more than 10 out of 80 DDR genes (Fig. 1a, Supplementary Table 4). Next, we evaluated the effect of these miRNAs on OS of ovarian cancer patients based on a set of TCGA data including 570 ovarian cancer patients receiving platinum treatment. We showed that patients with high levels of miR-211 exhibited a significantly longer survival time than patients with low levels of miR-211 by using either best survival separation model (Fig. 1b, *p* < 0.001) or median value separation model (Supplementary Fig. 1A, *p* < 0.01) based on the expression of miR-211. Whereas other detected miRNAs (miR-204, let-7 and miR-421) failed to show significant association with the prognosis of ovarian cancer patients (Supplementary Fig. 1B, D, *p* > 0.05). Thus, we focused our study on miR-211 and enlarged the research scope of DDR genes targeted by miR-211 to explore the molecular mechanism.

We also performed a univariate analysis using Cox proportional hazard regression to define the clinical application value of miR-211. Low miR-211 expression was associated with worse OS, and high expression was associated with improved OS (high vs. low by median, HR of death = 0.70, 95% CI = 0.56–0.89, *p* < 0.003). In addition, miR-211 correlated with the consequence of platinum treatment in ovarian cancer patients (Fig. 1c). As shown in Fig. 1d, patients exhibiting a complete response to platinum were significantly more represented in the group with high miR-211 levels than in the low level group (254/345 vs. 63/108).

In addition, given the wealth of transcriptome datasets of ovarian cancer, we also analyzed the prognostic value of miR-211 target genes. A total of 321 targets of miR-211 were identified according to miRTarBase (Supplementary Table 5). Of these, 23 targets were revealed to exhibit a significant inverse correlation with miR-211 expression in the TCGA dataset (Supplementary Fig. 2 and Supplementary Table 6). We then investigated whether these 23 target genes of miR-211 are prognostic in ovarian cancer. For this purpose, we collected 2 TCGA datasets and 14 transcriptome datasets of ovarian cancer from previous studies, including primary tumors in a total of 2532 patients. We first normalized the expression of these 23 genes using Z-scores and calculated their average expression for each primary tumor. For each dataset, patients were then divided into two equal groups based on the average expression of the 23 genes. In most datasets, patients with high expression of miR-211 target genes exhibited the worse survival (Fig. 1e). We performed a meta-analysis across the 14 datasets and identified a significant association (high vs. low miR-211 targets expression, HR of death = 1.17, 95% CI = 1.05–1.30, *p* < 0.01), which may further confirm that high miR-211 expression is likely associated with favorable OS. These results indicated directly or indirectly that miR-211 is an indicator for a favorable prognosis of ovarian cancer probably via influencing the response to chemotherapy.

### miR-211 promotes DNA damage induced by carboplatin via inhibiting DDR genes

**Fig. 2:**
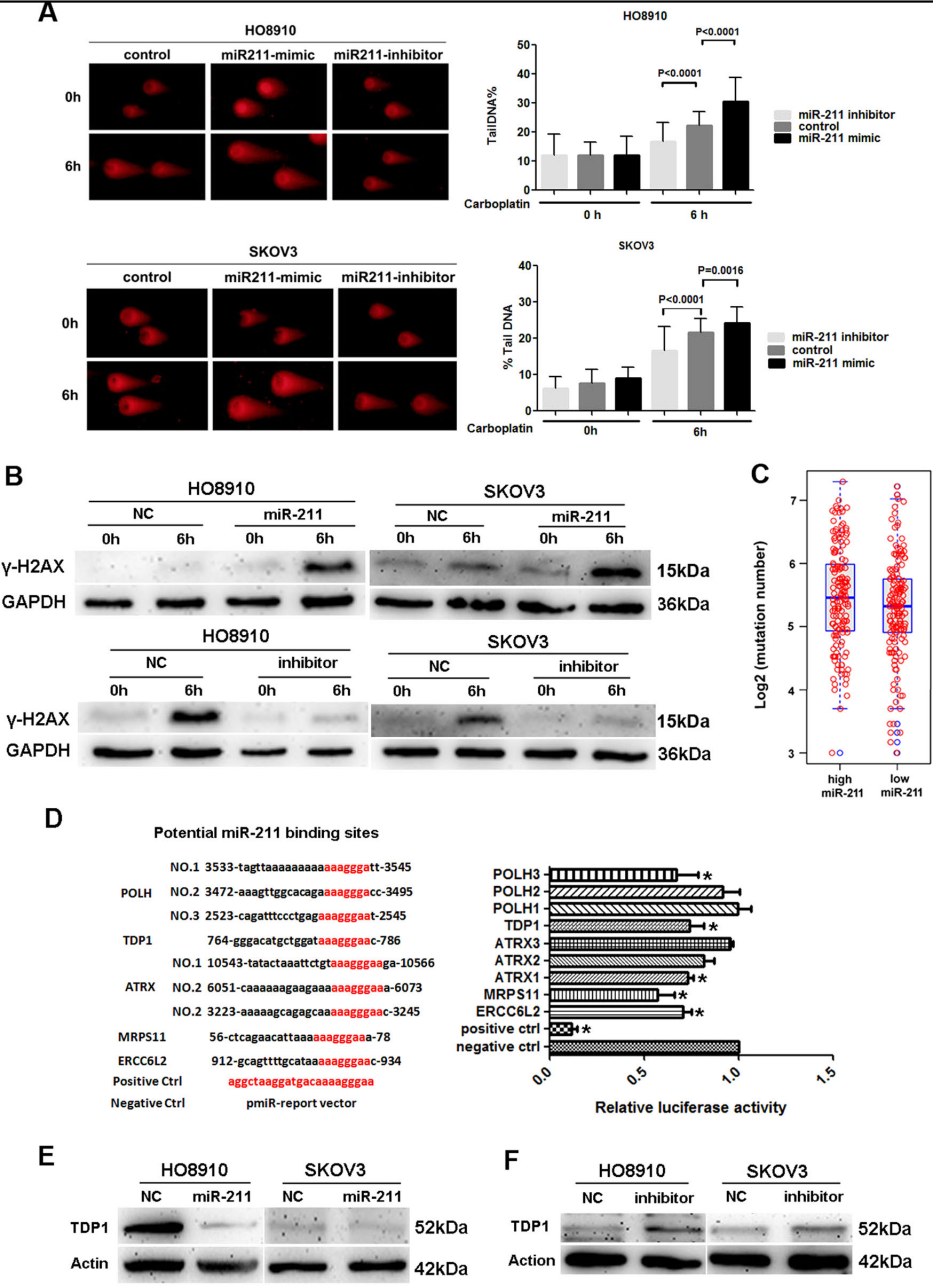
miR-211 promoted DNA damage induced by carboplatin via inhibiting DDR genes. **a** Carboplatin induced more significant DNA damage in ovarian cancer cells transfected with miR-211, whereas miR-211 inhibitor impaired the function of carboplatin as assessed by the comet assay. **b** Transfection of miR-211 increased the expression of γ-H2AX in HO8910 and SKOV3 cells treated with 0.25 mM carboplatin in the early stage, whereas amiR-211 inhibitor decreased γ-H2AX expression in these cells treated with 0.5 mM carboplatin. **c** Patients with higher levels of miR-211 exhibited an increased mutation frequency. **d** The dual luciferase assay confirmed that five DDR genes were targets of miR-211. **e** Transfection of miR-211 inhibited TDP1 expression in HO8910 and SKOV3 cells as assessed by western blot. **f** Suppression of miR-211 using inhibitor upregulated TDP1 expression in ovarian cancer cells

To explore the related mechanism, we first assessed the effect of miR-211 on DNA damage induced by carboplatin using comet assay and by detecting γ-H2AX, a marker for the degree of early DNA damage. The results indicated that transfection of miR-211, contrary to the miR-211 inhibitor, resulted in more significant DNA damage in ovarian cancer cells treated with carboplatin according to the comet assay (Fig. 2a). Our results indicated that γ-H2AX induced by carboplatin was upregulated in miR-211-overexpressing cells but was downregulated in cells treated with miR-211 inhibitor (Fig. 2b). Given the effect of miR-211 on DNA damage, we assessed the correlation between miR-211 level and genome instability of ovarian cancer using a set of mutation data from TCGA. Patients with higher miR-211 levels exhibited an increased frequency of mutation (Fig. 2c). Since mutations in the BRCA1/2 genes, involved in DDR, are common in high-grade serous ovarian cancer, and increase mutational burden, thus the correlation between miR-211 expression and mutation burden might be influenced by the BRCA mutation status. To investigate if miR-211 increasing mutational burden of ovarian cancer samples is associated with BRCA mutations, we used the most updated mutation data downloaded from GDSC to get the somatic mutation of BRCA1/2 from a previous study^27^. In total, we collected 75 samples with either somatic or germline mutations of BRCA1/2 (Supplementary Table 7). We confirmed that ovarian cancers with BRCA mutations have significantly higher mutation burden than those cancers without BRCA mutations (Supplementary Fig. 3A, Wilcox rank sum test, *p* < 0.0001). However, we did not find any significant difference of miR-211 expression between BRCA mutant and WT ovarian cancer samples (Supplementary Fig. 3B, *p* > 0.05). Moreover, the partial correlation between miR-211 expression and mutation burden is also significant after correcting for BRCA mutation status (Supplementary Fig. 3C, Spearman’s rho = 0.114, *p* = 0.043), suggesting the BRCA mutation is not likely to affect the association between miR-211 expression and mutations burden strongly. Finally, we separated the ovarian cancer samples by the median value of miR-211 expression, and observed a relatively longer survival time in miR-211 high group regardless of BRCA mutational status (Supplementary Fig. 3D), although only a marginal significance is achieved due to the reduced number of samples in each case. These results indicated that miR-211 could promote DNA damage induced by carboplatin.

To explore the molecular mechanisms by which miR-211 inhibits DDR, we performed GO function enrichment analysis on the predicted target genes of miR-211, and 20 genes were identified that were related to DDR (Supplementary Table 8). We verified the regulatory function of miR-211 on these target genes using a dual luciferase assay, and five DDR genes (POLH, TDP1, ATRX, MRPS11, and ERCC6L2) were ultimately verified as the targets of miR-211 (Fig. 2d). Furthermore, we confirmed the regulatory effect of miR-211 on DDR genes, which was represented by the relationship between miR-211 and TDP1. The results indicated that miR-211 overexpression inhibited TDP1 expression, whereas suppressing miR-211 upregulated TDP1 expression in ovarian cancer cells (Fig. 2e, f). These results indicated that miR-211 can inhibit the expression of DDR genes, revealing the potential mechanism by which miR-211 promotes chemosensitivity in ovarian cancer.

### miR-211 enhanced the chemosensitivity of ovarian cancer to carboplatin

**Fig. 3:**
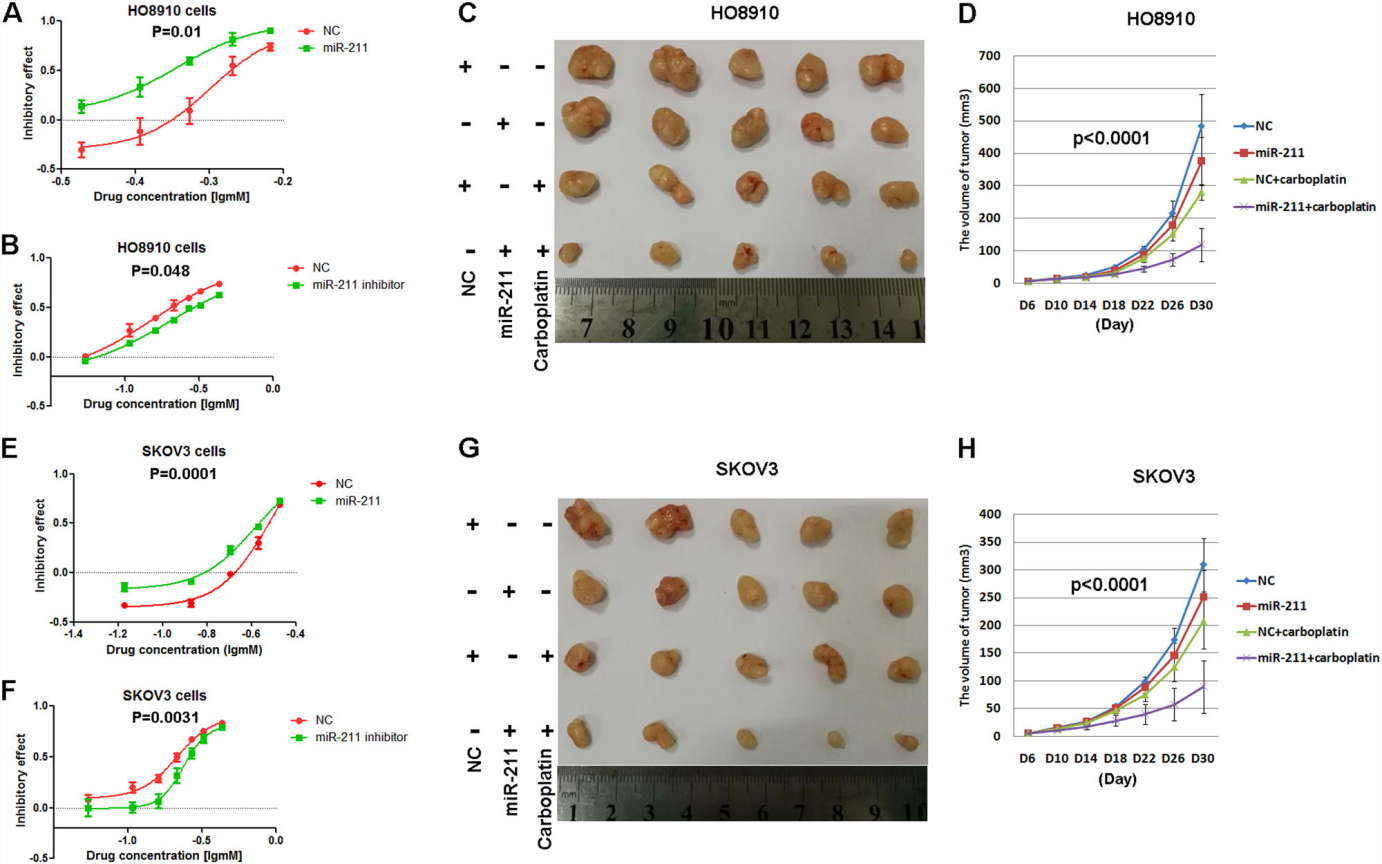
miR-211 improved the chemosensitivity of ovarian cancer to carboplatin. **a**, **e** Transfection of miR-211 improved the sensitivity of HO8910 and SKOV3 to carboplatin according to cell viability assays. **b**, **f** Inhibiting miR-211 decreased the sensitivity of HO8910 and SKOV3 to carboplatin. **c**, **g** Intratumoral injection of miR-211 agomiR significantly inhibited subcutaneous tumor growth in mice treated with carboplatin via intraperitoneal administration. **d**, **h** Growth curve of HO8910 and SKOV3 subcutaneous xenograft tumors

Next, we detected the effect of miR-211 on carboplatin treatment in vivo and in vitro. We found that miR-211 improved the sensitivity of ovarian cancer cells to carboplatin, whereas the sensitivity was decreased when miR-211 activity was inhibited in both HO8910 (Fig. 3a, b) and SKOV3 (Fig. 3e, f) cells. Compared with the NC, intratumoral injection of agomiR miR-211 significantly inhibited subcutaneous tumor growth in mice treated with carboplatin by intraperitoneal administration (Fig. 3c, d for HO8910, 3g and 3h for SKOV3). These results indicated that miR-211 increased the chemosensitivity of ovarian cancer to carboplatin.

### TDP1 is the key target mediating the function of miR-211

**Fig. 4:**
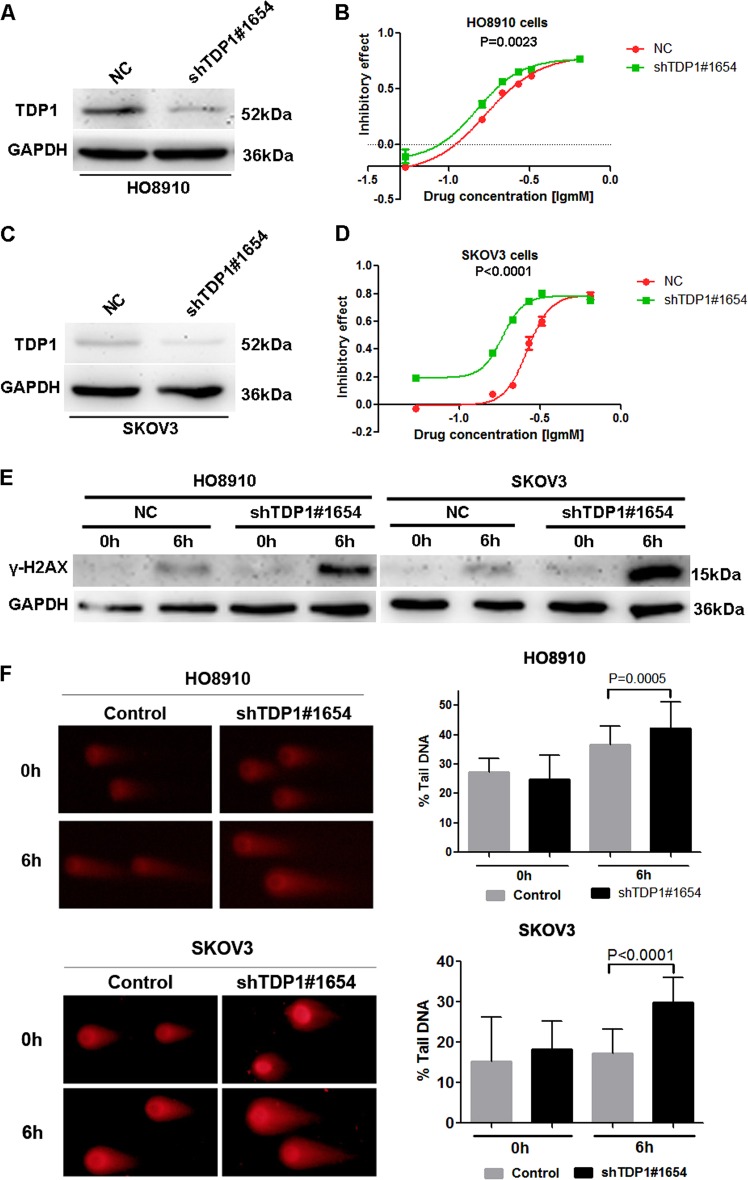
TDP1 is the key target mediating the function of miR-211. **a**, **c** The shRNA technique effectively blocked the expression of TDP1 in HO8910 and SKOV3 cells. **b**, **d** Downregulation of TDP1 facilitated the sensitivity of HO8910 and SKOV3 cells to carboplatin. **e** Knockdown of TDP1 resulted in the upregulation of γ-H2AX in HO8910 and SKOV3 cells treated with carboplatin. **f**, **g** The comet assay indicated that TDP1 knockdown resulted in more significant DNA damage in HO8910 and SKOV3 cells treated with carboplatin

To verify that TDP1 is one of the key molecules that mediates the function of miR-211, we further detected the role of TDP1 in chemosensitivity and DNA damage in ovarian cancer cells. The results indicated that both HO8910 (Fig. 4a, b) and SKOV3 (Fig. 4c, d) cells were more sensitive to carboplatin when TDP1 was knocked down by shRNA. In addition, knockdown of TDP1 also resulted in more significant DNA damage as reflected by increased γ-H2AX (Fig. 4e) and more tail DNA in the comet assay (Fig. 4f) in HO8910 and SKOV3 cells treated with carboplatin. The results indicated that TDP1 deficiency promoted DNA damage and increased the chemosensitivity of ovarian cancer cells to carboplatin.

### miR-211 improved the neoadjuvant chemosensitivity of ovarian cancer patients

**Fig. 5:**
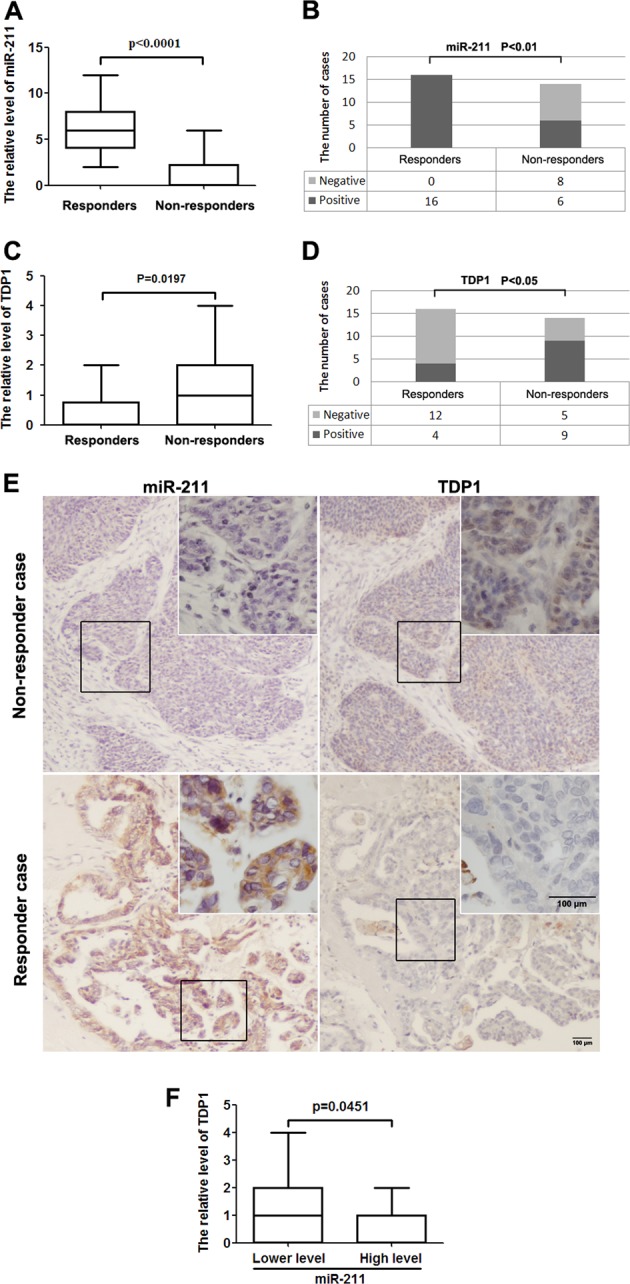
miR-211 improved neoadjuvant chemosensitivity by targeting the DDR gene. **a** The levels of miR-211 in the responders were significantly increased compared with those in non-responders receiving neoadjuvant chemotherapy. **b** The positive rate of miR-211 was also increased in the responders compared with that in the non-responders. **c** TDP1 levels were reduced in the responders compared with those in the non-responders. **d** The positive rate of TDP1 was also reduced in the responders. **e** miR-211 expression was negatively associated with TDP1 expression in samples from ovarian cancer patients. **f** TDP1 levels were reduced in patients with high miR-211 levels but increased in patients with low miR-211 levels

Finally, we assessed the correlation between miR-211 or TDP1 levels and the effect of neoadjuvant chemotherapy by immunohistochemistry and in situ hybridization. The levels of miR-211 in the responders were significantly increased compared with those in the non-responders (Fig. 5a). The positive rate of miR-211 was also increased in the responders (Fig. 5b). In contrast, TDP1 levels were decreased in the responders (Fig. 5c), and the positive rate of TDP1 was significantly reduced in the responders compared with the non-responders, as shown in Fig. 5d. In addition, TDP1 levels were reduced in the group with increased miR-211 levels, but increased in the group with lower miR-211 levels (Fig. 5e, f). These results further proved that increased miR-211 or decreased TDP1 can improve the chemosensitivity of ovarian cancer and provide additional proof for the inhibitory effect of miR-211 on TDP1.

## Discussion

Chemotherapy is one of the most important therapeutics for malignancies, including ovarian cancer. Platinum-based agents are first-line chemotherapies for ovarian cancer and improve the prognosis of ovarian cancer to a great extent^28,29^. However, not all patients respond to this treatment. Resistance to platinum appears in ~30% of newly diagnosed patients and almost all patients with recurrent ovarian cancer^30–32^. Primary or acquired resistance is the major obstacle for chemotherapy.

miRNAs are a type of small noncoding RNA that can posttranscriptionally regulate gene expression and function in multiple biological processes^13^. Increasing evidence has suggested that miRNAs are involved in the regulation of platinum chemosensitivity, and some of these miRNAs exhibit significant potential by acting as biomarkers to predict chemotherapeutic efficiency^33–35^. DDR is induced when the cells are treated with platinum^36^, and the induction of DDR is one of the most important mechanisms for platinum resistance^37–40^. In this study, we found that miR-211 exhibited significant potential to bind and inhibit DDR genes. The following analysis revealed that miR-211 levels were positively correlated with the response to platinum in ovarian cancer, and that increased miR-211 faciliated the OS of ovarian cancer patients. Based on these data, we hypothesized that miR-211 might affect the prognosis of ovarian cancer by inhibiting DDR genes and subsequently improving the chemotherapeutic effect.

To verify our hypothesis, we detected the effect of miR-211 on DNA damage and the potential molecular mechanism by which miR-211 regulated DDR genes. The results suggested that miR-211 facilitated platinum-induced DNA damage and exhibited a potential regulatory effect on five DDR genes, including POLH, TDP1, ATRX, MRPS11, and ERCC6L2. TDP1 plays an important role in repairing double-strand DNA breaks^41^, and it is upregulated in multiple malignancies and involved in the regulation of chemosensitivity^42,43^. Moreover, TDP1 encodes Tyrosyl-DNA phosphodiesterase and there are several inhibitors of TDP1 have been developed and tested for sensitizer of chemotherapy based on DNA damage reagents^44–46^. Consequently, targeting TDP1 might be a promising strategy to sensitize cancer cells to platinum in clinic. Taken together, we chose TDP1 as the representation to reflect the association between miR-211 and DDR genes. In this study, TDP1 knockdown enhanced the sensitivity of cancer cells to carboplatin and resulted in more significant DNA damage. The association between miR-211 and TDP1 was also supported by clinical data from patients who received neoadjuvant chemotherapy. Thus, we concluded that miR-211 can improve the sensitivity of ovarian cancer cells to platinum by targeting DDR genes, thereby further influencing the prognosis of ovarian cancer.

Currently, aberrant expression and function of miR-211 in cancer have gained considerable attention, and increasing data have indicated that miR-211 likely functions as a tumor suppressor^47–49^. However, no study has revealed the relationship between miR-211 and DDR in cancer chemotherapy to date. It has been demonstrated that miR-211 is downregulated in ovarian cancer, and restoration of miR-211 inhibits cell proliferation by suppressing cyclin D1 and CDK6 expression^50^. In addition to proliferation and metastasis, miR-211 is also involved in the regulation of chemosensitivity in cancer. For example, Asuthkar et al. reported that miR-211 promoted the sensitivity of glioblastoma multiforme (GBM) to temozolomide (chemotherapy drug) by targeting MMP-9^51^. Recently, the interaction between lncRNA and miR-211 was implicated in chemoresistance. LncRNA KCNQ1OT1 was found to promoted chemoresistance by acting as a competing endogenous RNA (ceRNA) to bind miR-211-5p in tongue squamous cell carcinoma (TSCC)^52^. Reciprocal repression has been observed between lncRNA NEAT1 and miR-211 and results in upregulation of HMGA2 and subsequent 5-Fu resistance in breast cancer^53^.

In conclusion, this study indicated that miR-211 influences the prognosis of ovarian cancer patients by enhancing the chemosensitivity of cancer cells to platinum via inhibiting the expression of DDR genes and preventing the DNA damage repair process. Thus, miR-211 might represent an effective intervention target to improve platinum chemosensitivity in ovarian cancer. In addition, miR-211 also exhibits significant potential as a marker to predict the response to chemotherapy in ovarian cancer patients.

## Supplementary information

Supplementary Figures (1–3)

Supplementary Tables (1–4, 6–8)

Supplementary Table 5
