# Supplementary material for: miR-211 facilitates platinum chemosensitivity by blocking the DNA damage response (DDR) in ovarian cancer
Source: Cell Death Dis. 2019 Jun 24;10(7):495. doi: 10.1038/s41419-019-1715-x (PMC6591289; doi:10.1038/s41419-019-1715-x)
Supplement: Supplementary file 1 — Supplementary Figures (1–3) [file 41419_2019_1715_MOESM1_ESM.pdf]

Supplementary Figure 1.

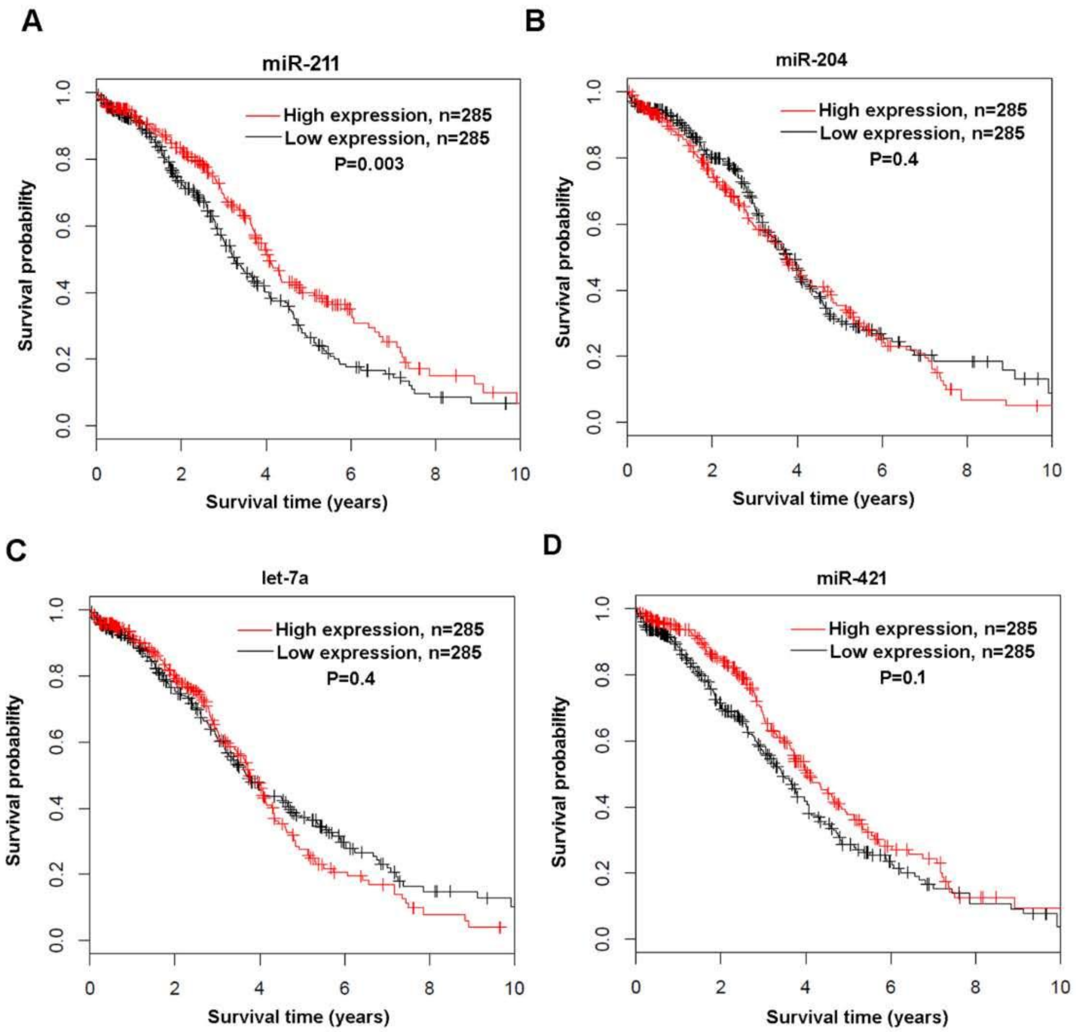

Supplementary Figure 1. Analyzing the association between miRNAs expression and prognosis of ovarian cancer patients. miR-211 (A) is positively correlated with OS of ovarian cancer, whereas miR-204 (B), let-7 (C) and miR-421 (D) fails to show significant association with OS of ovarian cancer patients.

Supplementary Figure 2.

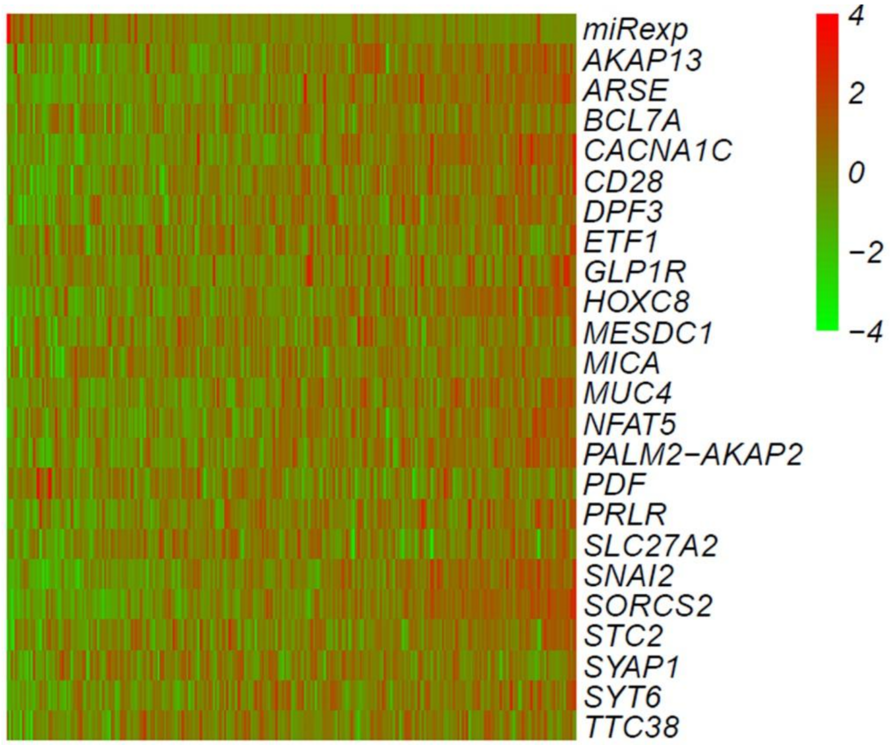

Supplementary Figure 2. Heatmap of the expression of miR-211 and 23 target genes negatively correlated with miR-211.

Supplementary Figure 3

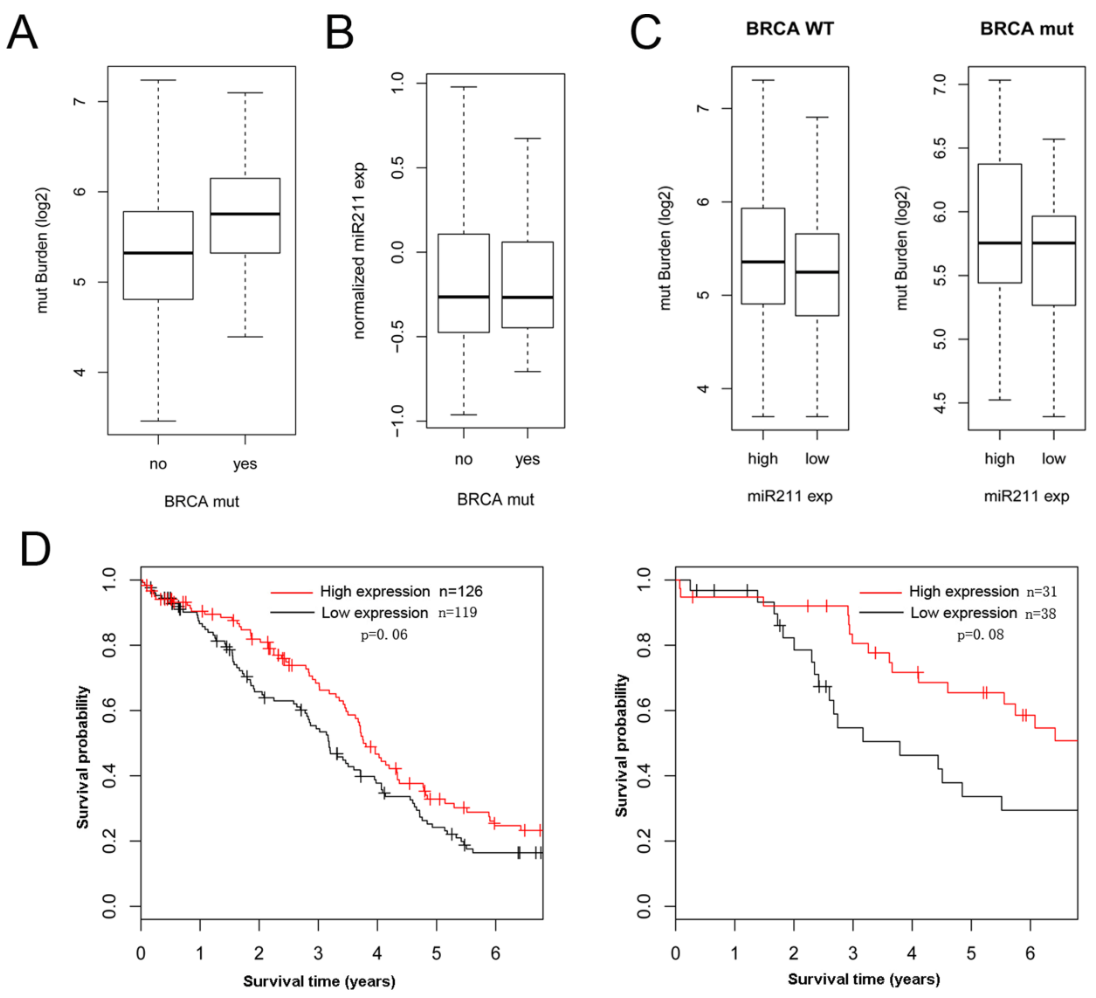

Supplementary Figure 3. Evaluating the influence of BRCA mutation status on the association between miR-211 levels and mutation burden of ovarian cancer samples. (A) Ovarian cancers with BRCA mutations have significantly higher mutation burden compared with those cancers without BRCA mutations. (B) There is no difference for miR-211 expression between BRCA mutant and WT ovarian cancer samples. (C) The partial correlation between miR-211 expression and mutation burden is also significant after correcting for BRCA mutation status. (D) miR-211 level is associated with the favorable outcome of ovarian cancer regardless of BRCA mutational status.
