# Supplementary material for: miR-211 facilitates platinum chemosensitivity by blocking the DNA damage response (DDR) in ovarian cancer
Source: Cell Death Dis. 2019 Jun 24;10(7):495. doi: 10.1038/s41419-019-1715-x (PMC6591289; doi:10.1038/s41419-019-1715-x)
Supplement: Supplementary file 2 — Supplementary Tables (1–4, 6–8) [file 41419_2019_1715_MOESM2_ESM.pdf]

Supplementary data

Supplementary Table 1. The clinical characteristics of ovarian cancer patients

Characteristics	Total (n=30)	Responders (n=16)	Non-responders (n=14)	P value
Age (y, mean ± SD)	51.2 ±7.04	52.5 ±7.03	48.64 ±6.34	>0.05
FIGO stage				>0.05
I	6 (20%)	5 (16.7%)	1 (3.3%)	
III	24 (80%)	11 (36.7%)	13 (43.3%)	
Differentiation				>0.05
moderately	26 (86.7%)	14 (46.6%)	12 (40%)	
poorly	4 (13.3%)	2 (6.7%)	2 (6.7%)	
Tumor size (diameter, cm)				>0.05
≤1cm	2 (6.7%)	1 (3.3%)	1 (3.3%)	
>1cm	28 (93.3)	15 (50%)	13 (43.4%)	

Supplementary Table 2. Primers used to construct luciferase reporter plasmids, and sequence used in this study

**Genes**	**Primers (5'-3')**
Positive control	F:*cgcgt*AGGCGAAGGATGACAAAGGGAA*a*
	R: *agctt*TTCCCTTTGTCATCCTTCGCCT*a*
CHEK1	F1:*cgcgt*tcttctgttggatg**aaaggga**ta*a*
	R1:*agctt*TATCCCTTTCATCCAACAGAAGA*a*
	F2:*cgcgt*acttcctgaagatt**aaagggaa**g*a*
	R2:*agctt*CTTCCCTTTAATCTTCAGGAAGT*a*
RAD23B	F:*cgcgt*gggtgggaggggag**aaagggaa**c*a*
	R:*agctt*GTTCCCTTTCTCCCCTCCCACCC*a*
RAD51	F:*cgcgt*ctcagctatgtagc**aaagggaa**t*a*
	R:*agctt*ATTCCCTTTGCTACATAGCTGAG*a*
RINT1	F:*cgcgt*agtactcttgtttg**aaaggga**gc*a*
	R:*agctt*GCTCCCTTTCAAACAAGAGTACT*a*
TRIAP1	F:*cgcgt*aactaatgctctga**aaaggga**tc*a*
	R:*agctt*GATCCCTTTTCAGAGCATTAGTT*a*
UPF1	F:*cgcgt*ttgcgcccctgtgg**aaaggga**tc*a*
	R:*agctt*GATCCCTTTCCACAGGGGCGCAA*a*
RAD52	F:*cgcgt*ttggactctgtcac**aaaggga**ct*a*
	R:*agctt*AGTCCCTTTGTGACAGAGTCCAA*a*
ALKBH1	F1:*cgcgt*cctgggtggtcttc**aaaggga**tg*a*
	R1:*agctt*CATCCCTTTGAAGACCACCCAGG*a*
	F2:*cgcgt*actaccatcttcct**aaaggga**ta*a*
	R2:*agctt*TATCCCTTTAGGAAGATGGTAGT*a*
ATRX	F1:*cgcgt*aaaaagcagagcaaa**aagggaa**c*a*
	R1:*agctt*GTTCCCTTTTTGCTCTGCTTTTT*a*
	F2:*cgcgt*caaaaaagaagaaa**aaagggaa**a*a*
	R2:*agctt*TTTCCCTTTTTTCTTCTTTTTTG*a*
	F3:*cgcgt*atactaaattctgt**aaaggga**ga*a*
	R3:*agctt*TCTCCCTTTACAGAATTTAGTAT*a*
ERCC6L2	F:*cgcgt*gcagttttgcataa**aaagggaa**c*a*
	R:*agctt*GTTCCCTTTTTATGCAAAACTGC*a*
CUL4B	F:*cgcgt*gttctgttacactg**aaaggga**tg*a*
	R:*agctt*CATCCCTTTCAGTGTAACAGAAC*a*
CCNA2	F:*cgcgt*ggatctctaatgaa**aaaggga**tg*a*
	R:*agctt*CATCCCTTTTTCATTAGAGATCC*a*
GTF2H3	F:*cgcgt*acgtagctttggaa**aaaggga**ct*a*
	R:*agctt*AGTCCCTTTTTCCAAAGCTACGT*a*
HMGB1	F:*cgcgt*cctgatgcagcaaa**aaaggga**gt*a*
	R:*agctt*ACTCCCTTTTTTGCTGCATCAGG*a*
INT3	F:*cgcgt*tatcctgacagagc**aaaggga**gt*a*

	R:*agctt*ACTCCCTTTGCTCTGTCAGGATA*a*
DDB2	F:*cgcgt*aacccaccttcatc**aaaggga**tt*a*
	R:*agctt*AATCCCTTTGATGAAGGTGGGTT*a*
POLH	F1:*cgcgt*cagatttccctgag**aaagggaa**t*a*
	R1:*agctt*ATTCCCTTTCTCAGGGAAATCTG*a*
	F2:*cgcgt*aaagttggcacaga**aaaggga**cc*a*
	R2:*agctt*GGTCCCTTTTCTGTGCCAACTTT*a*
	F3:*cgcgt*tagttaaaaaaaaa**aaaggga**tt*a*
	R3:*agctt*AATCCCTTTTTTTTTTTTAACTA*a*
SSRP1	F:*cgcgt*gaaaatgatttaat**aaagggaa**c*a*
	R:*agctt*GTTCCCTTTATTAAATCATTTTC*a*
TDP1	F:*cgcgt*gggacatgctggat**aaagggaa**c*a*
	R:*agctt*GTTCCCTTTATCCAGCATGTCCC*a*
MRPS11	F: *cgcgt*ctcagaacattaaa**aaagggaa**a*a*
	R: *agctt*TTTCCCTTTTTTAATGTTCTGAG*a*
miR-211-5p mimics	Sense 5’-UUCCCUUUGUCAUCCUUCGCCU-3’
	Antisense 5’-GCGAAGGAUGACAAAGGGAAUU-3’
miR-211 inhibitor	5’-AGGCGAAGGAUGACAAAGGGAA-3’
miR-211 antisense probe	5’-AGGCGAAGGATGACAAAGGGAA-3’
ShTDP1	AGTTTCAAAGTGAAACAGAAG

Supplementary Table 3. 80 DDR genes that exhibited a response to platinum drugs.

Gene symbol	DDR pathway	Gene symbol	DDR pathway	Gene symbol	DDR pathway	Gene symbol	DDR pathway
CETN2	NER	POLK	TLS	ERCC2	NER	MDC1	ATM
DDB2	NER	REV3L	TLS	RAD52	FA/HR	RAD1	ATM
ERCC8	NER	RAD50	FA/HR	XPA	NER	ERCC6	NER
RAD17	ATM	CHEK2	ATM	POLI	TLS	BRIP1	FA/HR
H2AFX	ATM	RAD54B	FA/HR	TOPBP1	ATM	USP1	FA/HR
HLTF	TLS	FBXO18	TLS	ATR	ATM	FANCE	FA/HR
ERCC1	NER	FANCB	FA/HR	UBE2N	ATM	RAD51C	FA/HR
UBE2B	TLS	NBN	FA/HR	ERCC5	NER	SHFM1	FA/HR
RAD9A	ATM	FANCG	FA/HR	ATM	ATM	FANCD2	FA/HR
C17orf70	FA/HR	CLSPN	ATM	RNF8	ATM	MAD2L2	TLS
BRCA2	FA/HR	FANCL	FA/HR	ERCC3	NER	REV1	TLS
FANCI	FA/HR	ATRIP	ATM	DDB1	NER	SHPRH	TLS
RBBP8	FA/HR	HUS1	ATM	FANCC	FA/HR	MUS81	FA/HR
UBE2V2	TLS	RAD18	TLS	XRCC3	FA/HR	MRE11A	FA/HR
FANCA	FA/HR	POLH	TLS	RAD23B	NER	RAD51	FA/HR
PALB2	FA/HR	CHEK1	ATM	RAD23A	NER	XRCC2	FA/HR
TP53BP1	ATM	WDR48	FA/HR	FANCM	FA/HR	UBE2I	TLS
RAD54L	FA/HR	TOP2A	ATM	XAB2	NER	ERCC4	NER
BLM	FA/HR	BRCA1	FA/HR	TP53	ATM	RAD51L1	FA/HR
C19orf40	FA/HR	FANCF	FA/HR	XPC	NER	RAD51L3	FA/HR

Supplementary Table 4. The top 9 miRNAs targeting DDR genes responding to platinum

miRNA	DDR genes responding to platinum
miR-211/204	RAD51, RAD52, ATM, HLTF, RAD23B, WDR48, DDB2, POLH, UBE2B, CHEK1, REV3E, UBE2V2, PALB2, FANCL
Let-7	MDC1, WDR48, RAD23B, RAD9A, ERCC5, RNF8, FANCD2, FANCC, TOPBP1, ERCC6, TP53, UBE2B, TOP2A
miR-421	SHFM1, RAD50, FANCE, RAD1, RAD54L, XRCC2, REV3E, CHEK1, UBE2B, RAD17, TP53
miR-301b	SHFM1, RBBP8, RAD51B, FANCM, NBN, ERCC4, POLK, FANCA, ERCC6, POLH
miR-146a	BLM, FANCI, BRCA1, ATR, CHEK1, TOP2A
miR-505	SHPRH, REV3E, CHEK1, RAD17, SHFM1
miR-324	UBE2I, RAD17, UBE2B, MDC1
miR-320	FANCF, RAD9A, RAD23B, HLTF
miR-193a	UBE2N, WDR48, MDC1

Supplementary Table 6. 23 target genes are significantly negatively correlated with miR-211.

Target Gene	Experiments	Functions	Ref.(PMID)	P value
AKAP13	HITS-CLIP	Anchors cAMP- dependent protein kinase.	19536157	0.0498477
ARSE	HITS-CLIP	The composition of cartilage and bone matrix during development.	23824327	0.0461715
BCL7A	HITS-CLIP	Not clear.	23824327	0.0040596
CACNA1C	PAR-CLIP	Muscle contraction/ hormone or neuro- transmitter release/ Gene expression /Cell motility,division, death	22100165	0.0333576
CD28	HITS-CLIP	T-cell activation/Cell proliferation/Cytokine production/T-cell survival	23824327	0.0320574
DPF3	HITS-CLIP	Self-renewal or proliferative capacity of the multipotent neural stem cells/Dendrite growth/transcriptional activation and repression / development of heart and skeletal muscle.	23824327	0.0298730
ETF1	HITS-CLIP	Translational regulation	23824327	0.0450870
GLP1R	HITS-CLIP	A receptor for glucagon-like peptide 1	23824327	0.0382149
HOXC8	PAR-CLIP	Transcription factor	20371350	0.0163929
MESDC1	HITS-CLIP	Not clear	23824327	0.0005623
MICA	HITS-CLIP	Ligand for the KLRK-1/NKG2D receptor	23824327	0.0203057
MUC4	HITS-CLIP	Repression of apoptosis/Anti-adhesive properties/ Proliferation	23824327	0.0189769
PALM2-AKAP2	HITS-CLIP	Not Clear	23824327	0.0142971
PDF	HITS-CLIP	Removal of the formyl group from the N-terminal Met of newly synthesized proteins	23824327	0.0434386
PRLR	HITS-CLIP	A receptor for the anterior pituitary hormone prolactin/	23824327	0.0162198

		Prosurvival factor for spermatozoa		
SLC27A2	HITS-CLIP	Bile acid metabolism/Fatty acid metabolism	23824327	0.0041475
SNAI2	HITS-CLIP	Transcriptional repressor	23824327	0.0356045
SORCS2	PAR-CLIP	Not Clear	20371350	0.0495637
STC2	HITS-CLIP	Regulating calcium and phosphate homeostasis	23824327	0.0016057
SYAP1	HITS-CLIP	Not Clear	23824327	0.0426751
SYT6	HITS-CLIP	Ca(2+)-dependent exocytosis of secretory vesicles	23824327	0.0119142
TTC38	HITS-CLIP	Not Clear	23824327	2.10505e-06
NFAT5	Luciferase reporter assay, Microarray, qRT-PCR, Western blot	Transcription factor	24039954	0.0058981

Supplementary Table 7. 75 samples with either somatic or germline mutations of BRCA1/2

Samples	Genetic defect	Additional information
TCGA-04-1357	BRCA1 somatic mutation	Nonsense_Mutation
TCGA-13-0730	BRCA1 somatic mutation	Nonsense_Mutation
TCGA-13-0761	BRCA1 somatic mutation	Splice_Site
TCGA-13-0804	BRCA1 somatic mutation	Missense_Mutation
TCGA-13-1489	BRCA1 somatic mutation	Frame_Shift_Ins
TCGA-24-2035	BRCA1 somatic mutation	Frame_Shift_Del
TCGA-25-1625	BRCA1 somatic mutation	Nonsense_Mutation
TCGA-29-2427	BRCA1 somatic mutation	Nonsense_Mutation
TCGA-04-1331	BRCA2 somatic mutation	Nonsense_Mutation
TCGA-09-2050	BRCA2 somatic mutation	Nonsense_Mutation
TCGA-13-0792	BRCA2 somatic mutation	Missense_Mutation
TCGA-13-0885	BRCA2 somatic mutation	Frame_Shift_Del
TCGA-13-0890	BRCA2 somatic mutation	Frame_Shift_Del
TCGA-13-1481	BRCA2 somatic mutation	Frame_Shift_Del
TCGA-23-1030	BRCA2 somatic mutation	Missense_Mutation
TCGA-23-1120	BRCA2 somatic mutation	Frame_Shift_Del
TCGA-24-1103	BRCA2 somatic mutation	Missense_Mutation
TCGA-24-1555	BRCA2 somatic mutation	Frame_Shift_Del
TCGA-29-1693	BRCA2 somatic mutation	Frame_Shift_Del
TCGA-29-1762	BRCA2 somatic mutation	Nonsense_Mutation
TCGA-61-1903	BRCA2 somatic mutation	Missense_Mutation
TCGA-09-2051	BRCA1/2 germline mutation	Obtained from 21720365
TCGA-10-0931	BRCA1/2 germline mutation	Obtained from 21720365
TCGA-13-0887	BRCA1/2 germline mutation	Obtained from 21720365
TCGA-13-0893	BRCA1/2 germline mutation	Obtained from 21720365
TCGA-23-1122	BRCA1/2 germline mutation	Obtained from 21720365
TCGA-57-1582	BRCA1/2 germline mutation	Obtained from 21720365
TCGA-59-2348	BRCA1/2 germline mutation	Obtained from 21720365
TCGA-24-1470	BRCA1/2 germline mutation	Obtained from 21720365
TCGA-25-2401	BRCA1/2 germline mutation	Obtained from 21720365
TCGA-23-1118	BRCA1/2 germline mutation	Obtained from 21720365
TCGA-09-2045	BRCA1/2 germline mutation	Obtained from 21720365
TCGA-13-0883	BRCA1/2 germline mutation	Obtained from 21720365
TCGA-10-0927	BRCA1/2 germline mutation	Obtained from 21720365
TCGA-25-1318	BRCA1/2 germline mutation	Obtained from 21720365
TCGA-23-2077	BRCA1/2 germline mutation	Obtained from 21720365
TCGA-23-2079	BRCA1/2 germline mutation	Obtained from 21720365
TCGA-13-1512	BRCA1/2 germline mutation	Obtained from 21720365
TCGA-23-1023	BRCA1/2 germline mutation	Obtained from 21720365
TCGA-09-1669	BRCA1/2 germline mutation	Obtained from 21720365

TCGA-13-0889	BRCA1/2 germline mutation	Obtained from 21720365
TCGA-23-2081	BRCA1/2 germline mutation	Obtained from 21720365
TCGA-04-1356	BRCA1/2 germline mutation	Obtained from 21720365
TCGA-23-2078	BRCA1/2 germline mutation	Obtained from 21720365
TCGA-25-2392	BRCA1/2 germline mutation	Obtained from 21720365
TCGA-13-1408	BRCA1/2 germline mutation	Obtained from 21720365
TCGA-13-0802	BRCA1/2 germline mutation	Obtained from 21720365
TCGA-61-2008	BRCA1/2 germline mutation	Obtained from 21720365
TCGA-13-0903	BRCA1/2 germline mutation	Obtained from 21720365
TCGA-10-0937	BRCA1/2 germline mutation	Obtained from 21720365
TCGA-23-1027	BRCA1/2 germline mutation	Obtained from 21720365
TCGA-24-2298	BRCA1/2 germline mutation	Obtained from 21720365
TCGA-61-2109	BRCA1/2 germline mutation	Obtained from 21720365
TCGA-04-1367	BRCA1/2 germline mutation	Obtained from 21720365
TCGA-13-1498	BRCA1/2 germline mutation	Obtained from 21720365
TCGA-59-2351	BRCA1/2 germline mutation	Obtained from 21720365
TCGA-13-0766	BRCA1/2 germline mutation	Obtained from 21720365
TCGA-24-2288	BRCA1/2 germline mutation	Obtained from 21720365
TCGA-13-0913	BRCA1/2 germline mutation	Obtained from 21720365
TCGA-25-1634	BRCA1/2 germline mutation	Obtained from 21720365
TCGA-04-1336	BRCA1/2 germline mutation	Obtained from 21720365
TCGA-24-2293	BRCA1/2 germline mutation	Obtained from 21720365
TCGA-13-0886	BRCA1/2 germline mutation	Obtained from 21720365
TCGA-24-1562	BRCA1/2 germline mutation	Obtained from 21720365
TCGA-24-1463	BRCA1/2 germline mutation	Obtained from 21720365
TCGA-13-1499	BRCA1/2 germline mutation	Obtained from 21720365
TCGA-13-0793	BRCA1/2 germline mutation	Obtained from 21720365
TCGA-24-2280	BRCA1/2 germline mutation	Obtained from 21720365
TCGA-25-1318	BRCA1/2 germline mutation	Obtained from 21720365
TCGA-57-1584	BRCA1/2 germline mutation	Obtained from 21720365
TCGA-13-1512	BRCA1/2 germline mutation	Obtained from 21720365
TCGA-13-0726	BRCA1/2 germline mutation	Obtained from 21720365
TCGA-24-0975	BRCA1/2 germline mutation	Obtained from 21720365
TCGA-24-1417	BRCA1/2 germline mutation	Obtained from 21720365
TCGA-24-1555	BRCA1/2 germline mutation	Obtained from 21720365
TCGA-24-2024	BRCA1/2 germline mutation	Obtained from 21720365
TCGA-23-1026	BRCA1/2 germline mutation	Obtained from 21720365
TCGA-25-2404	BRCA1/2 germline mutation	Obtained from 21720365

Supplementary Table 8. 20 targets of miR-211 were associated with DNA damage response

Gene name	Pathways	Potential binding sites within target mRNAs
CHEK1	ATM	1316-1322 (ORF); 2272-2279 (ORF)
RAD23B	NER	2200-2207(3'UTR)
RAD51	HR	1732-1739(3'UTR)
RAD52	HR	1431-1437(3'UTR)
RINT1	DNA damage checkpoint	1457-1463(ORF)
TRIAP1	DNA damage response	711-717(3'UTR)
UPF1	DNA binding and genome stability maintanance	1431-1437(ORF);4855-4860(3'UTR)
ALKBH1	Repair of alkylation damaged DNA	788-794(ORF);2306-2312(3'UTR)
TRX	DNA damage response	3237-3243/6065-6072 (ORF) 10558-10564(3'UTR)
ERCC6L2	DNA repair	925-932(ORF)
CUL4B	DNA repair	3566-3572(3'UTR)
CCNA2	DNA damage checkpoint	2690-2696(3'UTR)
GTF2H3	DNA repair	1429-1435(3'UTR)
HMGB1	DNA binding and repair	679-685(ORF);2420-2425(3'UTR)
INT3	DNA damage response	1125-1131(ORF)
TDP1	BER	777-784(ORF)
DDB2	NER	646-652(ORF)
POLH	TLS	2537-2544/3486-3492/3547-3553(3'UTR)
SSRP1	HR	2796-2803(3'UTR)
MRPS11	DNA damage response	56-78 (5'UTR)
