# Supplementary material for: miR-211 facilitates platinum chemosensitivity by blocking the DNA damage response (DDR) in ovarian cancer
Source: Cell Death Dis. 2019 Jun 24;10(7):495. doi: 10.1038/s41419-019-1715-x (PMC6591289; doi:10.1038/s41419-019-1715-x)
Supplement: Supplementary file 3 — Supplementary Table 5 [file 41419_2019_1715_MOESM3_ESM.pdf]

Supplementary Table 5. The 321 targets of miR-211 identified according to miRTarBase

miRTarBase ID	miRNA	Species (miRNA)	Target Gene
MIRT636431	hsa-miR-211-5p	Homo sapiens	1-Mar
MIRT618848	hsa-miR-211-5p	Homo sapiens	A1CF
MIRT643804	hsa-miR-211-5p	Homo sapiens	ABCC12
MIRT624863	hsa-miR-211-5p	Homo sapiens	ABHD15
MIRT539417	hsa-miR-211-5p	Homo sapiens	ADAT2
MIRT720868	hsa-miR-211-5p	Homo sapiens	ADCY5
MIRT660887	hsa-miR-211-5p	Homo sapiens	ADCYAP1R1
MIRT717451	hsa-miR-211-5p	Homo sapiens	ADORA3
MIRT660848	hsa-miR-211-5p	Homo sapiens	AFF4
MIRT648240	hsa-miR-211-5p	Homo sapiens	AGAP1
MIRT683218	hsa-miR-211-5p	Homo sapiens	AGO2
MIRT669584	hsa-miR-211-5p	Homo sapiens	AK2
MIRT662352	hsa-miR-211-5p	Homo sapiens	AKAP13
MIRT662352	hsa-miR-211-5p	Homo sapiens	AKAP13
MIRT660777	hsa-miR-211-5p	Homo sapiens	AKAP2
MIRT632076	hsa-miR-211-5p	Homo sapiens	ALDH1A2
MIRT660770	hsa-miR-211-5p	Homo sapiens	ALDH6A1
MIRT660700	hsa-miR-211-5p	Homo sapiens	AMOTL2
MIRT624758	hsa-miR-211-5p	Homo sapiens	ANGPTL2
MIRT660683	hsa-miR-211-5p	Homo sapiens	ANKFY1
MIRT647179	hsa-miR-211-5p	Homo sapiens	ANKRD45
MIRT054445	hsa-miR-211-5p	Homo sapiens	AP1S2
MIRT054445	hsa-miR-211-5p	Homo sapiens	AP1S2
MIRT617471	hsa-miR-211-5p	Homo sapiens	AP5B1
MIRT624684	hsa-miR-211-5p	Homo sapiens	ARAP2
MIRT638968	hsa-miR-211-5p	Homo sapiens	ARHGAP6
MIRT185715	hsa-miR-211-5p	Homo sapiens	ARNTL2
MIRT615211	hsa-miR-211-5p	Homo sapiens	ARSE
MIRT631538	hsa-miR-211-5p	Homo sapiens	AS3MT
MIRT724362	hsa-miR-211-5p	Homo sapiens	ASGR1
MIRT713974	hsa-miR-211-5p	Homo sapiens	ASIC4
MIRT645432	hsa-miR-211-5p	Homo sapiens	ATF6B
MIRT613805	hsa-miR-211-5p	Homo sapiens	ATP6AP1L
MIRT167969	hsa-miR-211-5p	Homo sapiens	ATXN1
MIRT641312	hsa-miR-211-5p	Homo sapiens	ATXN7
MIRT641469	hsa-miR-211-5p	Homo sapiens	B4GALNT3
MIRT660361	hsa-miR-211-5p	Homo sapiens	BACH2
MIRT650426	hsa-miR-211-5p	Homo sapiens	BBS9
MIRT489334	hsa-miR-211-5p	Homo sapiens	BCAN
MIRT489334	hsa-miR-211-5p	Homo sapiens	BCAN
MIRT489334	hsa-miR-211-5p	Homo sapiens	BCAN
MIRT489334	hsa-miR-211-5p	Homo sapiens	BCAN
MIRT438781	hsa-miR-211-5p	Homo sapiens	BCL2
MIRT615113	hsa-miR-211-5p	Homo sapiens	BCL7A
MIRT626402	hsa-miR-211-5p	Homo sapiens	BID
MIRT660284	hsa-miR-211-5p	Homo sapiens	BLOC1S5
MIRT660229	hsa-miR-211-5p	Homo sapiens	BMPR1A
MIRT613279	hsa-miR-211-5p	Homo sapiens	BRD4
MIRT660081	hsa-miR-211-5p	Homo sapiens	BZW1

MIRT707108	hsa-miR-211-5p	Homo sapiens	C11orf74
MIRT660060	hsa-miR-211-5p	Homo sapiens	C12orf5
MIRT649274	hsa-miR-211-5p	Homo sapiens	C17orf64
MIRT619999	hsa-miR-211-5p	Homo sapiens	C1orf64
MIRT617362	hsa-miR-211-5p	Homo sapiens	C21orf62
MIRT644147	hsa-miR-211-5p	Homo sapiens	C4orf3
MIRT646888	hsa-miR-211-5p	Homo sapiens	C6orf132
MIRT659933	hsa-miR-211-5p	Homo sapiens	CACHD1
MIRT444933	hsa-miR-211-5p	Homo sapiens	CACNA1C
MIRT624479	hsa-miR-211-5p	Homo sapiens	CACNG8
MIRT659901	hsa-miR-211-5p	Homo sapiens	CAMK2G
MIRT659861	hsa-miR-211-5p	Homo sapiens	CAPRIN1
MIRT526584	hsa-miR-211-5p	Homo sapiens	CCDC43
MIRT659710	hsa-miR-211-5p	Homo sapiens	CCDC93
MIRT641337	hsa-miR-211-5p	Homo sapiens	CCR5
MIRT662215	hsa-miR-211-5p	Homo sapiens	CCRL2
MIRT659683	hsa-miR-211-5p	Homo sapiens	CD28
MIRT719257	hsa-miR-211-5p	Homo sapiens	CD44
MIRT095329	hsa-miR-211-5p	Homo sapiens	CDC23
MIRT619800	hsa-miR-211-5p	Homo sapiens	CDC42EP4
MIRT624381	hsa-miR-211-5p	Homo sapiens	CDH4
MIRT006257	hsa-miR-211-5p	Homo sapiens	CDH5
MIRT609199	hsa-miR-211-5p	Homo sapiens	CDH8
MIRT614151	hsa-miR-211-5p	Homo sapiens	CEP97
MIRT614151	hsa-miR-211-5p	Homo sapiens	CEP97
MIRT642780	hsa-miR-211-5p	Homo sapiens	CHCHD3
MIRT618150	hsa-miR-211-5p	Homo sapiens	CHCHD5
MIRT642147	hsa-miR-211-5p	Homo sapiens	CHORDC1
MIRT640839	hsa-miR-211-5p	Homo sapiens	CHRDL1
MIRT621382	hsa-miR-211-5p	Homo sapiens	CLNK
MIRT615190	hsa-miR-211-5p	Homo sapiens	CLUAP1
MIRT646347	hsa-miR-211-5p	Homo sapiens	CPT1B
MIRT006462	hsa-miR-211-5p	Homo sapiens	CREB5
MIRT636590	hsa-miR-211-5p	Homo sapiens	DCAF7
MIRT054455	hsa-miR-211-5p	Homo sapiens	DDIT3
MIRT659098	hsa-miR-211-5p	Homo sapiens	DENR
MIRT715237	hsa-miR-211-5p	Homo sapiens	DHODH
MIRT658968	hsa-miR-211-5p	Homo sapiens	DNAJB5
MIRT658968	hsa-miR-211-5p	Homo sapiens	DNAJB5
MIRT624096	hsa-miR-211-5p	Homo sapiens	DNAJC16
MIRT618424	hsa-miR-211-5p	Homo sapiens	DNAJC30
MIRT615254	hsa-miR-211-5p	Homo sapiens	DPF2
MIRT617323	hsa-miR-211-5p	Homo sapiens	DPF3
MIRT646198	hsa-miR-211-5p	Homo sapiens	DUSP10
MIRT504460	hsa-miR-211-5p	Homo sapiens	EID2B
MIRT280118	hsa-miR-211-5p	Homo sapiens	ELMSAN1
MIRT006463	hsa-miR-211-5p	Homo sapiens	ELOVL6
MIRT646524	hsa-miR-211-5p	Homo sapiens	ELP2
MIRT646524	hsa-miR-211-5p	Homo sapiens	ELP2
MIRT658622	hsa-miR-211-5p	Homo sapiens	ENAH

MIRT638755	hsa-miR-211-5p	Homo sapiens	EPHA4
MIRT628244	hsa-miR-211-5p	Homo sapiens	ETF1
MIRT703848	hsa-miR-211-5p	Homo sapiens	ETV3
MIRT658438	hsa-miR-211-5p	Homo sapiens	FAM167B
MIRT658333	hsa-miR-211-5p	Homo sapiens	FAM83F
MIRT658263	hsa-miR-211-5p	Homo sapiens	FAXC
MIRT647957	hsa-miR-211-5p	Homo sapiens	FBXO31
MIRT611807	hsa-miR-211-5p	Homo sapiens	FCRL4
MIRT616716	hsa-miR-211-5p	Homo sapiens	FEM1B
MIRT658120	hsa-miR-211-5p	Homo sapiens	FNBP1L
MIRT099067	hsa-miR-211-5p	Homo sapiens	FOXC1
MIRT615984	hsa-miR-211-5p	Homo sapiens	FSTL4
MIRT668238	hsa-miR-211-5p	Homo sapiens	FZD8
MIRT658023	hsa-miR-211-5p	Homo sapiens	G3BP2
MIRT623852	hsa-miR-211-5p	Homo sapiens	GALNT1
MIRT620589	hsa-miR-211-5p	Homo sapiens	GALNT10
MIRT623837	hsa-miR-211-5p	Homo sapiens	GAN
MIRT640772	hsa-miR-211-5p	Homo sapiens	GLP1R
MIRT648254	hsa-miR-211-5p	Homo sapiens	GP2
MIRT639760	hsa-miR-211-5p	Homo sapiens	GPR45
MIRT623733	hsa-miR-211-5p	Homo sapiens	GTDC1
MIRT649525	hsa-miR-211-5p	Homo sapiens	GTF3C3
MIRT716093	hsa-miR-211-5p	Homo sapiens	HARS
MIRT657487	hsa-miR-211-5p	Homo sapiens	HCAR2
MIRT668000	hsa-miR-211-5p	Homo sapiens	HCFC2
MIRT646404	hsa-miR-211-5p	Homo sapiens	HLA-DRB1
MIRT618805	hsa-miR-211-5p	Homo sapiens	HLA-DRB5
MIRT718175	hsa-miR-211-5p	Homo sapiens	HLCS
MIRT438707	hsa-miR-211-5p	Homo sapiens	HMX1
MIRT657357	hsa-miR-211-5p	Homo sapiens	HNRNPA2B1
MIRT248293	hsa-miR-211-5p	Homo sapiens	HOXC8
MIRT248293	hsa-miR-211-5p	Homo sapiens	HOXC8
MIRT623660	hsa-miR-211-5p	Homo sapiens	HRK
MIRT647671	hsa-miR-211-5p	Homo sapiens	HTR1F
MIRT054454	hsa-miR-211-5p	Homo sapiens	IGF2R
MIRT054449	hsa-miR-211-5p	Homo sapiens	IGFBP5
MIRT054449	hsa-miR-211-5p	Homo sapiens	IGFBP5
MIRT536708	hsa-miR-211-5p	Homo sapiens	IKZF2
MIRT536708	hsa-miR-211-5p	Homo sapiens	IKZF2
MIRT007023	hsa-miR-211-5p	Homo sapiens	IL11
MIRT007023	hsa-miR-211-5p	Homo sapiens	IL11
MIRT487493	hsa-miR-211-5p	Homo sapiens	IL1F10
MIRT487493	hsa-miR-211-5p	Homo sapiens	IL1F10
MIRT645827	hsa-miR-211-5p	Homo sapiens	INADL
MIRT644057	hsa-miR-211-5p	Homo sapiens	IQCE
MIRT567132	hsa-miR-211-5p	Homo sapiens	IRF2BP2
MIRT162736	hsa-miR-211-5p	Homo sapiens	ITPR1
MIRT725294	hsa-miR-211-5p	Homo sapiens	KANSL1
MIRT005943	hsa-miR-211-5p	Homo sapiens	KCNMA1
MIRT623489	hsa-miR-211-5p	Homo sapiens	KCTD11

MIRT642906	hsa-miR-211-5p	Homo sapiens	KIAA0754
MIRT641626	hsa-miR-211-5p	Homo sapiens	KIAA1244
MIRT656956	hsa-miR-211-5p	Homo sapiens	KIAA1324L
MIRT656928	hsa-miR-211-5p	Homo sapiens	KIAA1462
MIRT648773	hsa-miR-211-5p	Homo sapiens	KLHL40
MIRT656794	hsa-miR-211-5p	Homo sapiens	KNTC1
MIRT616906	hsa-miR-211-5p	Homo sapiens	LINC00598
MIRT448697	hsa-miR-211-5p	Homo sapiens	LIPC
MIRT719818	hsa-miR-211-5p	Homo sapiens	LRRC4
MIRT656611	hsa-miR-211-5p	Homo sapiens	LRRC55
MIRT526373	hsa-miR-211-5p	Homo sapiens	LSAMP
MIRT722272	hsa-miR-211-5p	Homo sapiens	LURAP1
MIRT708671	hsa-miR-211-5p	Homo sapiens	LY6G6D
MIRT709770	hsa-miR-211-5p	Homo sapiens	LY6G6F
MIRT054448	hsa-miR-211-5p	Homo sapiens	M6PR
MIRT198273	hsa-miR-211-5p	Homo sapiens	MAFG
MIRT645022	hsa-miR-211-5p	Homo sapiens	MAGEB4
MIRT438782	hsa-miR-211-5p	Homo sapiens	MAP2K1
MIRT683213	hsa-miR-211-5p	Homo sapiens	MBNL1
MIRT656409	hsa-miR-211-5p	Homo sapiens	MCTP1
MIRT656362	hsa-miR-211-5p	Homo sapiens	MDFI
MIRT656362	hsa-miR-211-5p	Homo sapiens	MDFI
MIRT713623	hsa-miR-211-5p	Homo sapiens	MED8
MIRT656308	hsa-miR-211-5p	Homo sapiens	MESDC1
MIRT656250	hsa-miR-211-5p	Homo sapiens	MEX3A
MIRT635071	hsa-miR-211-5p	Homo sapiens	MICA
MIRT556122	hsa-miR-211-5p	Homo sapiens	MIER3
MIRT619602	hsa-miR-211-5p	Homo sapiens	MKKS
MIRT053791	hsa-miR-211-5p	Homo sapiens	MMP9
MIRT722164	hsa-miR-211-5p	Homo sapiens	MRPS15
MIRT649769	hsa-miR-211-5p	Homo sapiens	MRPS27
MIRT649769	hsa-miR-211-5p	Homo sapiens	MRPS27
MIRT513815	hsa-miR-211-5p	Homo sapiens	MTA3
MIRT646744	hsa-miR-211-5p	Homo sapiens	MUC4
MIRT656047	hsa-miR-211-5p	Homo sapiens	MYLK4
MIRT667319	hsa-miR-211-5p	Homo sapiens	MYO18A
MIRT643658	hsa-miR-211-5p	Homo sapiens	MYOCD
MIRT655991	hsa-miR-211-5p	Homo sapiens	MYRF
MIRT054453	hsa-miR-211-5p	Homo sapiens	NFAT5
MIRT639063	hsa-miR-211-5p	Homo sapiens	NFATC1
MIRT655848	hsa-miR-211-5p	Homo sapiens	NGDN
MIRT655822	hsa-miR-211-5p	Homo sapiens	NOTCH1
MIRT664819	hsa-miR-211-5p	Homo sapiens	NOX5
MIRT655763	hsa-miR-211-5p	Homo sapiens	NPTX1
MIRT438079	hsa-miR-211-5p	Homo sapiens	NUAK1
MIRT623049	hsa-miR-211-5p	Homo sapiens	NUDT3
MIRT712684	hsa-miR-211-5p	Homo sapiens	NUDT7
MIRT715043	hsa-miR-211-5p	Homo sapiens	NUPL2
MIRT620042	hsa-miR-211-5p	Homo sapiens	ODF4
MIRT655619	hsa-miR-211-5p	Homo sapiens	ONECUT3

MIRT504152	hsa-miR-211-5p	Homo sapiens	OPN5
MIRT504152	hsa-miR-211-5p	Homo sapiens	OPN5
MIRT655542	hsa-miR-211-5p	Homo sapiens	PADI2
MIRT655542	hsa-miR-211-5p	Homo sapiens	PADI2
MIRT655432	hsa-miR-211-5p	Homo sapiens	PALM2-AKAP2
MIRT625842	hsa-miR-211-5p	Homo sapiens	PAQR7
MIRT054450	hsa-miR-211-5p	Homo sapiens	PDE3A
MIRT622864	hsa-miR-211-5p	Homo sapiens	PDF
MIRT343781	hsa-miR-211-5p	Homo sapiens	PHF13
MIRT643292	hsa-miR-211-5p	Homo sapiens	PHKG1
MIRT655094	hsa-miR-211-5p	Homo sapiens	PI4K2A
MIRT630664	hsa-miR-211-5p	Homo sapiens	PLAG1
MIRT654993	hsa-miR-211-5p	Homo sapiens	PLCG1
MIRT483170	hsa-miR-211-5p	Homo sapiens	PLCXD3
MIRT483170	hsa-miR-211-5p	Homo sapiens	PLCXD3
MIRT638421	hsa-miR-211-5p	Homo sapiens	PNRC1
MIRT650771	hsa-miR-211-5p	Homo sapiens	POP4
MIRT640921	hsa-miR-211-5p	Homo sapiens	POU2F1
MIRT521792	hsa-miR-211-5p	Homo sapiens	POU2F2
MIRT006355	hsa-miR-211-5p	Homo sapiens	POU3F2
MIRT654832	hsa-miR-211-5p	Homo sapiens	PPP1CC
MIRT654820	hsa-miR-211-5p	Homo sapiens	PPP3R1
MIRT626948	hsa-miR-211-5p	Homo sapiens	PRDM2
MIRT627372	hsa-miR-211-5p	Homo sapiens	PRICKLE4
MIRT666805	hsa-miR-211-5p	Homo sapiens	PRLR
MIRT646248	hsa-miR-211-5p	Homo sapiens	PRSS38
MIRT654603	hsa-miR-211-5p	Homo sapiens	PTPRT
MIRT546868	hsa-miR-211-5p	Homo sapiens	RAB10
MIRT546868	hsa-miR-211-5p	Homo sapiens	RAB10
MIRT654558	hsa-miR-211-5p	Homo sapiens	RAB11FIP4
MIRT654556	hsa-miR-211-5p	Homo sapiens	RAB14
MIRT006465	hsa-miR-211-5p	Homo sapiens	RAB22A
MIRT006465	hsa-miR-211-5p	Homo sapiens	RAB22A
MIRT654513	hsa-miR-211-5p	Homo sapiens	RAB5B
MIRT469640	hsa-miR-211-5p	Homo sapiens	RACGAP1
MIRT469640	hsa-miR-211-5p	Homo sapiens	RACGAP1
MIRT469640	hsa-miR-211-5p	Homo sapiens	RACGAP1
MIRT469640	hsa-miR-211-5p	Homo sapiens	RACGAP1
MIRT654340	hsa-miR-211-5p	Homo sapiens	RBM27
MIRT716155	hsa-miR-211-5p	Homo sapiens	RBM48
MIRT621143	hsa-miR-211-5p	Homo sapiens	RNF122
MIRT654177	hsa-miR-211-5p	Homo sapiens	RNMTL1
MIRT654162	hsa-miR-211-5p	Homo sapiens	RORB
MIRT618667	hsa-miR-211-5p	Homo sapiens	RPP40
MIRT054803	hsa-miR-211-5p	Homo sapiens	RRM2
MIRT642876	hsa-miR-211-5p	Homo sapiens	SAMD1
MIRT622355	hsa-miR-211-5p	Homo sapiens	SAMD5
MIRT722370	hsa-miR-211-5p	Homo sapiens	SEC31B
MIRT650841	hsa-miR-211-5p	Homo sapiens	SEMA4G
MIRT054446	hsa-miR-211-5p	Homo sapiens	SERINC3

MIRT653913	hsa-miR-211-5p	Homo sapiens	SERPINC1
MIRT662468	hsa-miR-211-5p	Homo sapiens	SERPINF2
MIRT512990	hsa-miR-211-5p	Homo sapiens	SEZ6L
MIRT512990	hsa-miR-211-5p	Homo sapiens	SEZ6L
MIRT627605	hsa-miR-211-5p	Homo sapiens	SFT2D2
MIRT057025	hsa-miR-211-5p	Homo sapiens	SGPL1
MIRT666430	hsa-miR-211-5p	Homo sapiens	SH2B3
MIRT649397	hsa-miR-211-5p	Homo sapiens	SH2D4A
MIRT646285	hsa-miR-211-5p	Homo sapiens	SH3BP5L
MIRT612505	hsa-miR-211-5p	Homo sapiens	SH3PXD2A
MIRT639168	hsa-miR-211-5p	Homo sapiens	SHOX2
MIRT617164	hsa-miR-211-5p	Homo sapiens	SLC16A5
MIRT107789	hsa-miR-211-5p	Homo sapiens	SLC1A1
MIRT646388	hsa-miR-211-5p	Homo sapiens	SLC22A6
MIRT649354	hsa-miR-211-5p	Homo sapiens	SLC27A2
MIRT653553	hsa-miR-211-5p	Homo sapiens	SLC38A7
MIRT622218	hsa-miR-211-5p	Homo sapiens	SLC39A11
MIRT666216	hsa-miR-211-5p	Homo sapiens	SLC39A9
MIRT725151	hsa-miR-211-5p	Homo sapiens	SLC43A1
MIRT653495	hsa-miR-211-5p	Homo sapiens	SLC43A2
MIRT627556	hsa-miR-211-5p	Homo sapiens	SMAD6
MIRT653325	hsa-miR-211-5p	Homo sapiens	SMOC1
MIRT636215	hsa-miR-211-5p	Homo sapiens	SNAI2
MIRT644653	hsa-miR-211-5p	Homo sapiens	SNX9
MIRT451888	hsa-miR-211-5p	Homo sapiens	SOD2
MIRT451888	hsa-miR-211-5p	Homo sapiens	SOD2
MIRT486360	hsa-miR-211-5p	Homo sapiens	SORCS2
MIRT486360	hsa-miR-211-5p	Homo sapiens	SORCS2
MIRT653232	hsa-miR-211-5p	Homo sapiens	SOS2
MIRT084444	hsa-miR-211-5p	Homo sapiens	SOWAHC
MIRT612417	hsa-miR-211-5p	Homo sapiens	SP1
MIRT617068	hsa-miR-211-5p	Homo sapiens	SPIB
MIRT645356	hsa-miR-211-5p	Homo sapiens	SPNS1
MIRT644582	hsa-miR-211-5p	Homo sapiens	SPOP
MIRT658826	hsa-miR-211-5p	Homo sapiens	SRCAP
MIRT653147	hsa-miR-211-5p	Homo sapiens	SRGAP1
MIRT054451	hsa-miR-211-5p	Homo sapiens	SSRP1
MIRT641504	hsa-miR-211-5p	Homo sapiens	STC2
MIRT644961	hsa-miR-211-5p	Homo sapiens	STEAP4
MIRT666034	hsa-miR-211-5p	Homo sapiens	STRBP
MIRT652952	hsa-miR-211-5p	Homo sapiens	SYAP1
MIRT652932	hsa-miR-211-5p	Homo sapiens	SYNGR1
MIRT666017	hsa-miR-211-5p	Homo sapiens	SYNJ2BP
MIRT617030	hsa-miR-211-5p	Homo sapiens	SYT6
MIRT532252	hsa-miR-211-5p	Homo sapiens	TBPL2
MIRT006464	hsa-miR-211-5p	Homo sapiens	TCF12
MIRT641842	hsa-miR-211-5p	Homo sapiens	TCF7L2
MIRT663296	hsa-miR-211-5p	Homo sapiens	TECPR2
MIRT053337	hsa-miR-211-5p	Homo sapiens	TGFB1
MIRT054452	hsa-miR-211-5p	Homo sapiens	TGFBR2

MIRT652683	hsa-miR-211-5p	Homo sapiens	TIAF1
MIRT497080	hsa-miR-211-5p	Homo sapiens	TLK2
MIRT648219	hsa-miR-211-5p	Homo sapiens	TM4SF20
MIRT497311	hsa-miR-211-5p	Homo sapiens	TMEFF2
MIRT618589	hsa-miR-211-5p	Homo sapiens	TMEM156
MIRT652445	hsa-miR-211-5p	Homo sapiens	TMEM236
MIRT639559	hsa-miR-211-5p	Homo sapiens	TMTC2
MIRT642183	hsa-miR-211-5p	Homo sapiens	TOR1AIP1
MIRT520547	hsa-miR-211-5p	Homo sapiens	TPPP
MIRT520547	hsa-miR-211-5p	Homo sapiens	TPPP
MIRT520547	hsa-miR-211-5p	Homo sapiens	TPPP
MIRT520547	hsa-miR-211-5p	Homo sapiens	TPPP
MIRT446103	hsa-miR-211-5p	Homo sapiens	TSC22D2
MIRT613766	hsa-miR-211-5p	Homo sapiens	TTC38
MIRT652064	hsa-miR-211-5p	Homo sapiens	TTC39B
MIRT130079	hsa-miR-211-5p	Homo sapiens	TXNIP
MIRT651976	hsa-miR-211-5p	Homo sapiens	UBE2J1
MIRT651902	hsa-miR-211-5p	Homo sapiens	UFD1L
MIRT661630	hsa-miR-211-5p	Homo sapiens	UGT2B28
MIRT650855	hsa-miR-211-5p	Homo sapiens	UNC13D
MIRT650914	hsa-miR-211-5p	Homo sapiens	VASH1
MIRT651767	hsa-miR-211-5p	Homo sapiens	VASP
MIRT570336	hsa-miR-211-5p	Homo sapiens	VAV3
MIRT570336	hsa-miR-211-5p	Homo sapiens	VAV3
MIRT570336	hsa-miR-211-5p	Homo sapiens	VAV3
MIRT570336	hsa-miR-211-5p	Homo sapiens	VAV3
MIRT620222	hsa-miR-211-5p	Homo sapiens	VN1R1
MIRT646764	hsa-miR-211-5p	Homo sapiens	WDR3
MIRT651554	hsa-miR-211-5p	Homo sapiens	WISP1
MIRT644041	hsa-miR-211-5p	Homo sapiens	WWC2
MIRT651481	hsa-miR-211-5p	Homo sapiens	WWC3
MIRT569806	hsa-miR-211-5p	Homo sapiens	XKR4
MIRT642949	hsa-miR-211-5p	Homo sapiens	XRRA1
MIRT639840	hsa-miR-211-5p	Homo sapiens	ZBTB20
MIRT609831	hsa-miR-211-5p	Homo sapiens	ZBTB22
MIRT720970	hsa-miR-211-5p	Homo sapiens	ZBTB43
MIRT715628	hsa-miR-211-5p	Homo sapiens	ZBTB8B
MIRT725063	hsa-miR-211-5p	Homo sapiens	ZCCHC24
MIRT489588	hsa-miR-211-5p	Homo sapiens	ZDHHC20
MIRT489588	hsa-miR-211-5p	Homo sapiens	ZDHHC20
MIRT489588	hsa-miR-211-5p	Homo sapiens	ZDHHC20
MIRT489588	hsa-miR-211-5p	Homo sapiens	ZDHHC20
MIRT489588	hsa-miR-211-5p	Homo sapiens	ZDHHC20
MIRT533009	hsa-miR-211-5p	Homo sapiens	ZFHX3
MIRT632191	hsa-miR-211-5p	Homo sapiens	ZKSCAN4
MIRT648387	hsa-miR-211-5p	Homo sapiens	ZNF22
MIRT651161	hsa-miR-211-5p	Homo sapiens	ZNF362
MIRT714745	hsa-miR-211-5p	Homo sapiens	ZNF385D
MIRT651131	hsa-miR-211-5p	Homo sapiens	ZNF398
MIRT651124	hsa-miR-211-5p	Homo sapiens	ZNF48

MIRT497627	hsa-miR-211-5p	Homo sapiens	ZNF576
MIRT661656	hsa-miR-211-5p	Homo sapiens	ZNF623
MIRT648805	hsa-miR-211-5p	Homo sapiens	ZNF689
MIRT646449	hsa-miR-211-5p	Homo sapiens	ZNF705A
MIRT644463	hsa-miR-211-5p	Homo sapiens	ZNF747
MIRT619978	hsa-miR-211-5p	Homo sapiens	ZSCAN22
MIRT611458	hsa-miR-211-5p	Homo sapiens	ZWILCH
MIRT531115	hsa-miR-211-5p	Homo sapiens	ZYG11B

Notes: There are 36 repeat targets, thus the total valid[illegible]

Target Gene (Entrez	Species (Targ	Experiments	Support Type	Reference
55016	Homo sapiens	HITS-CLIP	Functional MTI (Weak)	23824327
29974	Homo sapiens	HITS-CLIP	Functional MTI (Weak)	23824327
94160	Homo sapiens	HITS-CLIP	Functional MTI (Weak)	23824327
116236	Homo sapiens	HITS-CLIP	Functional MTI (Weak)	23824327
134637	Homo sapiens	PAR-CLIP	Functional MTI (Weak)	22012620
111	Homo sapiens	HITS-CLIP	Functional MTI (Weak)	19536157
117	Homo sapiens	HITS-CLIP	Functional MTI (Weak)	23824327
140	Homo sapiens	HITS-CLIP	Functional MTI (Weak)	19536157
27125	Homo sapiens	HITS-CLIP	Functional MTI (Weak)	23824327
116987	Homo sapiens	HITS-CLIP	Functional MTI (Weak)	23824327
27161	Homo sapiens	HITS-CLIP	Functional MTI (Weak)	23706177
204	Homo sapiens	HITS-CLIP	Functional MTI (Weak)	23824327
11214	Homo sapiens	HITS-CLIP	Functional MTI (Weak)	19536157
11214	Homo sapiens	HITS-CLIP	Functional MTI (Weak)	23824327
11217	Homo sapiens	HITS-CLIP	Functional MTI (Weak)	23824327
8854	Homo sapiens	HITS-CLIP	Functional MTI (Weak)	23824327
4329	Homo sapiens	HITS-CLIP	Functional MTI (Weak)	23824327
51421	Homo sapiens	HITS-CLIP	Functional MTI (Weak)	23824327
23452	Homo sapiens	HITS-CLIP	Functional MTI (Weak)	23824327
51479	Homo sapiens	HITS-CLIP	Functional MTI (Weak)	23824327
339416	Homo sapiens	HITS-CLIP	Functional MTI (Weak)	23824327
8905	Homo sapiens	Luciferase repor	Functional MTI	24039954
8905	Homo sapiens	HITS-CLIP	Functional MTI (Weak)	23824327
91056	Homo sapiens	HITS-CLIP	Functional MTI (Weak)	23824327
116984	Homo sapiens	HITS-CLIP	Functional MTI (Weak)	23824327
395	Homo sapiens	HITS-CLIP	Functional MTI (Weak)	23824327
56938	Homo sapiens	PAR-CLIP	Functional MTI (Weak)	20371350
415	Homo sapiens	HITS-CLIP	Functional MTI (Weak)	23824327
57412	Homo sapiens	HITS-CLIP	Functional MTI (Weak)	23824327
432	Homo sapiens	HITS-CLIP	Functional MTI (Weak)	19536157
55515	Homo sapiens	HITS-CLIP	Functional MTI (Weak)	19536157
1388	Homo sapiens	HITS-CLIP	Functional MTI (Weak)	23824327
92270	Homo sapiens	HITS-CLIP	Functional MTI (Weak)	23824327
6310	Homo sapiens	HITS-CLIP	Functional MTI (Weak)	23824327
6314	Homo sapiens	HITS-CLIP	Functional MTI (Weak)	23824327
283358	Homo sapiens	HITS-CLIP	Functional MTI (Weak)	23824327
60468	Homo sapiens	HITS-CLIP	Functional MTI (Weak)	23824327
27241	Homo sapiens	HITS-CLIP	Functional MTI (Weak)	23824327
63827	Homo sapiens	PAR-CLIP	Functional MTI (Weak)	20371350
63827	Homo sapiens	PAR-CLIP	Functional MTI (Weak)	21572407
63827	Homo sapiens	PAR-CLIP	Functional MTI (Weak)	22012620
63827	Homo sapiens	PAR-CLIP	Functional MTI (Weak)	23592263
596	Homo sapiens	qRT-PCR//Westerr	Functional MTI	24960059
605	Homo sapiens	HITS-CLIP	Functional MTI (Weak)	23824327
637	Homo sapiens	HITS-CLIP	Functional MTI (Weak)	23824327
63915	Homo sapiens	HITS-CLIP	Functional MTI (Weak)	23824327
657	Homo sapiens	HITS-CLIP	Functional MTI (Weak)	23824327
23476	Homo sapiens	HITS-CLIP	Functional MTI (Weak)	23824327
9689	Homo sapiens	HITS-CLIP	Functional MTI (Weak)	23824327

119710	Homo sapiens	HITS-CLIP	Functional MTI (Weak)	21572407
57103	Homo sapiens	HITS-CLIP	Functional MTI (Weak)	23824327
124773	Homo sapiens	HITS-CLIP	Functional MTI (Weak)	23824327
149563	Homo sapiens	HITS-CLIP	Functional MTI (Weak)	23824327
56245	Homo sapiens	HITS-CLIP	Functional MTI (Weak)	23824327
401152	Homo sapiens	HITS-CLIP	Functional MTI (Weak)	23824327
647024	Homo sapiens	HITS-CLIP	Functional MTI (Weak)	23824327
57685	Homo sapiens	HITS-CLIP	Functional MTI (Weak)	23824327
775	Homo sapiens	PAR-CLIP	Functional MTI (Weak)	22100165
59283	Homo sapiens	HITS-CLIP	Functional MTI (Weak)	23824327
818	Homo sapiens	HITS-CLIP	Functional MTI (Weak)	23824327
4076	Homo sapiens	HITS-CLIP	Functional MTI (Weak)	23824327
124808	Homo sapiens	PAR-CLIP	Functional MTI (Weak)	22012620
54520	Homo sapiens	HITS-CLIP	Functional MTI (Weak)	23824327
1234	Homo sapiens	HITS-CLIP	Functional MTI (Weak)	23824327
9034	Homo sapiens	HITS-CLIP	Functional MTI (Weak)	23824327
940	Homo sapiens	HITS-CLIP	Functional MTI (Weak)	23824327
960	Homo sapiens	HITS-CLIP	Functional MTI (Weak)	19536157
8697	Homo sapiens	PAR-CLIP	Functional MTI (Weak)	21572407
23580	Homo sapiens	HITS-CLIP	Functional MTI (Weak)	23824327
1002	Homo sapiens	HITS-CLIP	Functional MTI (Weak)	23824327
1003	Homo sapiens	Luciferase repor	Functional MTI	22235338
1006	Homo sapiens	HITS-CLIP	Functional MTI (Weak)	23824327
79598	Homo sapiens	HITS-CLIP	Functional MTI (Weak)	23313552
79598	Homo sapiens	HITS-CLIP	Functional MTI (Weak)	23824327
54927	Homo sapiens	HITS-CLIP	Functional MTI (Weak)	23824327
84269	Homo sapiens	HITS-CLIP	Functional MTI (Weak)	23824327
26973	Homo sapiens	HITS-CLIP	Functional MTI (Weak)	23824327
91851	Homo sapiens	HITS-CLIP	Functional MTI (Weak)	23824327
116449	Homo sapiens	HITS-CLIP	Functional MTI (Weak)	23824327
23059	Homo sapiens	HITS-CLIP	Functional MTI (Weak)	23824327
1375	Homo sapiens	HITS-CLIP	Functional MTI (Weak)	23824327
9586	Homo sapiens	Immunofluorescer	Functional MTI	22523078
10238	Homo sapiens	HITS-CLIP	Functional MTI (Weak)	23824327
1649	Homo sapiens	Luciferase repor	Functional MTI	24039954
8562	Homo sapiens	HITS-CLIP	Functional MTI (Weak)	23824327
1723	Homo sapiens	HITS-CLIP	Functional MTI (Weak)	19536157
25822	Homo sapiens	HITS-CLIP	Functional MTI (Weak)	19536157
25822	Homo sapiens	HITS-CLIP	Functional MTI (Weak)	23824327
23341	Homo sapiens	HITS-CLIP	Functional MTI (Weak)	23824327
84277	Homo sapiens	HITS-CLIP	Functional MTI (Weak)	23824327
5977	Homo sapiens	HITS-CLIP	Functional MTI (Weak)	23824327
8110	Homo sapiens	HITS-CLIP	Functional MTI (Weak)	23824327
11221	Homo sapiens	HITS-CLIP	Functional MTI (Weak)	23824327
126272	Homo sapiens	PAR-CLIP	Functional MTI (Weak)	23446348
91748	Homo sapiens	HITS-CLIP	Functional MTI (Weak)	23313552
79071	Homo sapiens	Immunofluorescer	Functional MTI	22523078
55250	Homo sapiens	HITS-CLIP	Functional MTI (Weak)	19536157
55250	Homo sapiens	HITS-CLIP	Functional MTI (Weak)	23824327
55740	Homo sapiens	HITS-CLIP	Functional MTI (Weak)	23824327

2043	Homo sapiens	HITS-CLIP	Functional MTI (Weak)		23824327
2107	Homo sapiens	HITS-CLIP	Functional MTI (Weak)		23824327
2117	Homo sapiens	HITS-CLIP	Functional MTI (Weak)		23313552
84734	Homo sapiens	HITS-CLIP	Functional MTI (Weak)		23824327
113828	Homo sapiens	HITS-CLIP	Functional MTI (Weak)		23824327
84553	Homo sapiens	HITS-CLIP	Functional MTI (Weak)		23824327
79791	Homo sapiens	HITS-CLIP	Functional MTI (Weak)		23824327
83417	Homo sapiens	HITS-CLIP	Functional MTI (Weak)		23824327
10116	Homo sapiens	HITS-CLIP	Functional MTI (Weak)		23824327
54874	Homo sapiens	HITS-CLIP	Functional MTI (Weak)		23824327
2296	Homo sapiens	PAR-CLIP	Functional MTI (Weak)		20371350
23105	Homo sapiens	HITS-CLIP	Functional MTI (Weak)		23824327
8325	Homo sapiens	HITS-CLIP	Functional MTI (Weak)		23824327
9908	Homo sapiens	HITS-CLIP	Functional MTI (Weak)		23824327
2589	Homo sapiens	HITS-CLIP	Functional MTI (Weak)		23824327
55568	Homo sapiens	HITS-CLIP	Functional MTI (Weak)		23824327
8139	Homo sapiens	HITS-CLIP	Functional MTI (Weak)		23824327
2740	Homo sapiens	HITS-CLIP	Functional MTI (Weak)		23824327
2813	Homo sapiens	HITS-CLIP	Functional MTI (Weak)		23824327
11250	Homo sapiens	HITS-CLIP	Functional MTI (Weak)		23824327
79712	Homo sapiens	HITS-CLIP	Functional MTI (Weak)		23824327
9330	Homo sapiens	HITS-CLIP	Functional MTI (Weak)		23824327
3035	Homo sapiens	HITS-CLIP	Functional MTI (Weak)		19536157
338442	Homo sapiens	HITS-CLIP	Functional MTI (Weak)		23824327
29915	Homo sapiens	HITS-CLIP	Functional MTI (Weak)		23824327
3123	Homo sapiens	HITS-CLIP	Functional MTI (Weak)		23824327
3127	Homo sapiens	HITS-CLIP	Functional MTI (Weak)		23824327
3141	Homo sapiens	HITS-CLIP	Functional MTI (Weak)		19536157
3166	Homo sapiens	Immunocytochemis	Functional MTI		24641951
3181	Homo sapiens	HITS-CLIP	Functional MTI (Weak)		23824327
3224	Homo sapiens	PAR-CLIP	Functional MTI (Weak)		20371350
3224	Homo sapiens	PAR-CLIP	Functional MTI (Weak)		21572407
8739	Homo sapiens	HITS-CLIP	Functional MTI (Weak)		23824327
3355	Homo sapiens	HITS-CLIP	Functional MTI (Weak)		23824327
3482	Homo sapiens	Luciferase repor	Functional MTI		24039954
3488	Homo sapiens	Luciferase repor	Functional MTI		24039954
3488	Homo sapiens	PAR-CLIP	Functional MTI (Weak)		20371350
22807	Homo sapiens	PAR-CLIP	Functional MTI (Weak)		22012620
22807	Homo sapiens	HITS-CLIP	Functional MTI (Weak)		23824327
3589	Homo sapiens	Luciferase repor	Functional MTI		22629385
3589	Homo sapiens	Luciferase repor	Functional MTI		24039954
84639	Homo sapiens	PAR-CLIP	Functional MTI (Weak)		20371350
84639	Homo sapiens	PAR-CLIP	Functional MTI (Weak)		23592263
10207	Homo sapiens	HITS-CLIP	Functional MTI (Weak)		23824327
23288	Homo sapiens	HITS-CLIP	Functional MTI (Weak)		23824327
359948	Homo sapiens	PAR-CLIP	Functional MTI (Weak)		20371350
3708	Homo sapiens	qRT-PCR//Westerr	Functional MTI		24960059
284058	Homo sapiens	HITS-CLIP	Functional MTI (Weak)		19536157
3778	Homo sapiens	Microarray//LacZ	Functional MTI		21072171
147040	Homo sapiens	HITS-CLIP	Functional MTI (Weak)		23824327

643314	Homo sapiens	HITS-CLIP	Functional MTI (Weak)	23824327
57221	Homo sapiens	HITS-CLIP	Functional MTI (Weak)	23824327
222223	Homo sapiens	HITS-CLIP	Functional MTI (Weak)	23824327
57608	Homo sapiens	HITS-CLIP	Functional MTI (Weak)	23824327
131377	Homo sapiens	HITS-CLIP	Functional MTI (Weak)	23824327
9735	Homo sapiens	HITS-CLIP	Functional MTI (Weak)	23824327
646982	Homo sapiens	HITS-CLIP	Functional MTI (Weak)	23824327
3990	Homo sapiens	PAR-CLIP	Functional MTI (Weak)	22100165
64101	Homo sapiens	HITS-CLIP	Functional MTI (Weak)	19536157
219527	Homo sapiens	HITS-CLIP	Functional MTI (Weak)	23824327
4045	Homo sapiens	PAR-CLIP	Functional MTI (Weak)	22012620
541468	Homo sapiens	HITS-CLIP	Functional MTI (Weak)	19536157
58530	Homo sapiens	HITS-CLIP	Functional MTI (Weak)	19536157
259215	Homo sapiens	HITS-CLIP	Functional MTI (Weak)	19536157
4074	Homo sapiens	Luciferase repor	Functional MTI	24039954
4097	Homo sapiens	HITS-CLIP	Functional MTI (Weak)	23824327
4115	Homo sapiens	HITS-CLIP	Functional MTI (Weak)	23824327
5604	Homo sapiens	qRT-PCR//Westerr	Functional MTI	24960059
4154	Homo sapiens	HITS-CLIP	Functional MTI (Weak)	23706177
79772	Homo sapiens	HITS-CLIP	Functional MTI (Weak)	23824327
4188	Homo sapiens	HITS-CLIP	Functional MTI (Weak)	19536157
4188	Homo sapiens	HITS-CLIP	Functional MTI (Weak)	23824327
112950	Homo sapiens	HITS-CLIP	Functional MTI (Weak)	19536157
59274	Homo sapiens	HITS-CLIP	Functional MTI (Weak)	23824327
92312	Homo sapiens	HITS-CLIP	Functional MTI (Weak)	23824327
100507436	Homo sapiens	HITS-CLIP	Functional MTI (Weak)	23824327
166968	Homo sapiens	PAR-CLIP	Functional MTI (Weak)	21572407
8195	Homo sapiens	HITS-CLIP	Functional MTI (Weak)	23824327
4318	Homo sapiens	Immunoblot//Immu	Functional MTI (Weak)	23183822
64960	Homo sapiens	HITS-CLIP	Functional MTI (Weak)	19536157
23107	Homo sapiens	HITS-CLIP	Functional MTI (Weak)	19536157
23107	Homo sapiens	HITS-CLIP	Functional MTI (Weak)	23824327
57504	Homo sapiens	PAR-CLIP	Functional MTI (Weak)	23446348
4585	Homo sapiens	HITS-CLIP	Functional MTI (Weak)	23824327
340156	Homo sapiens	HITS-CLIP	Functional MTI (Weak)	23824327
399687	Homo sapiens	HITS-CLIP	Functional MTI (Weak)	23824327
93649	Homo sapiens	HITS-CLIP	Functional MTI (Weak)	23824327
745	Homo sapiens	HITS-CLIP	Functional MTI (Weak)	23824327
10725	Homo sapiens	Luciferase repor	Functional MTI	24039954
4772	Homo sapiens	HITS-CLIP	Functional MTI (Weak)	23824327
25983	Homo sapiens	HITS-CLIP	Functional MTI (Weak)	23824327
4851	Homo sapiens	HITS-CLIP	Functional MTI (Weak)	23824327
79400	Homo sapiens	HITS-CLIP	Functional MTI (Weak)	23824327
4884	Homo sapiens	HITS-CLIP	Functional MTI (Weak)	23824327
9891	Homo sapiens	Luciferase repor	Functional MTI	23934065
11165	Homo sapiens	HITS-CLIP	Functional MTI (Weak)	23824327
283927	Homo sapiens	HITS-CLIP	Functional MTI (Weak)	19536157
11097	Homo sapiens	HITS-CLIP	Functional MTI (Weak)	19536157
146852	Homo sapiens	HITS-CLIP	Functional MTI (Weak)	23824327
390874	Homo sapiens	HITS-CLIP	Functional MTI (Weak)	23824327

221391	Homo sapiens	PAR-CLIP	Functional MTI (Weak)	22012620
221391	Homo sapiens	PAR-CLIP	Functional MTI (Weak)	23446348
11240	Homo sapiens	HITS-CLIP	Functional MTI (Weak)	19536157
11240	Homo sapiens	HITS-CLIP	Functional MTI (Weak)	23824327
445815	Homo sapiens	HITS-CLIP	Functional MTI (Weak)	23824327
164091	Homo sapiens	HITS-CLIP	Functional MTI (Weak)	23824327
5139	Homo sapiens	Luciferase repor	Functional MTI	24039954
64146	Homo sapiens	HITS-CLIP	Functional MTI (Weak)	23824327
148479	Homo sapiens	PAR-CLIP	Functional MTI (Weak)	20371350
5260	Homo sapiens	HITS-CLIP	Functional MTI (Weak)	23824327
55361	Homo sapiens	HITS-CLIP	Functional MTI (Weak)	23824327
5324	Homo sapiens	HITS-CLIP	Functional MTI (Weak)	23824327
5335	Homo sapiens	HITS-CLIP	Functional MTI (Weak)	23824327
345557	Homo sapiens	PAR-CLIP	Functional MTI (Weak)	22012620
345557	Homo sapiens	PAR-CLIP	Functional MTI (Weak)	23592263
10957	Homo sapiens	HITS-CLIP	Functional MTI (Weak)	23824327
10775	Homo sapiens	HITS-CLIP	Functional MTI (Weak)	23824327
5451	Homo sapiens	HITS-CLIP	Functional MTI (Weak)	23824327
5452	Homo sapiens	PAR-CLIP	Functional MTI (Weak)	23446348
5454	Homo sapiens	Luciferase repor	Functional MTI	21435193
5501	Homo sapiens	HITS-CLIP	Functional MTI (Weak)	23824327
5534	Homo sapiens	HITS-CLIP	Functional MTI (Weak)	23824327
7799	Homo sapiens	HITS-CLIP	Functional MTI (Weak)	23824327
29964	Homo sapiens	HITS-CLIP	Functional MTI (Weak)	23824327
5618	Homo sapiens	HITS-CLIP	Functional MTI (Weak)	23824327
339501	Homo sapiens	HITS-CLIP	Functional MTI (Weak)	23824327
11122	Homo sapiens	HITS-CLIP	Functional MTI (Weak)	23824327
10890	Homo sapiens	PAR-CLIP	Functional MTI (Weak)	20371350
10890	Homo sapiens	PAR-CLIP	Functional MTI (Weak)	21572407
84440	Homo sapiens	HITS-CLIP	Functional MTI (Weak)	23824327
51552	Homo sapiens	HITS-CLIP	Functional MTI (Weak)	23824327
57403	Homo sapiens	Immunofluorescer	Functional MTI	22523078
57403	Homo sapiens	Luciferase repor	Functional MTI	24039954
5869	Homo sapiens	HITS-CLIP	Functional MTI (Weak)	23824327
29127	Homo sapiens	PAR-CLIP	Functional MTI (Weak)	20371350
29127	Homo sapiens	PAR-CLIP	Functional MTI (Weak)	21572407
29127	Homo sapiens	PAR-CLIP	Functional MTI (Weak)	23446348
29127	Homo sapiens	PAR-CLIP	Functional MTI (Weak)	23592263
54439	Homo sapiens	HITS-CLIP	Functional MTI (Weak)	23824327
84060	Homo sapiens	HITS-CLIP	Functional MTI (Weak)	19536157
79845	Homo sapiens	HITS-CLIP	Functional MTI (Weak)	23824327
55178	Homo sapiens	HITS-CLIP	Functional MTI (Weak)	23824327
6096	Homo sapiens	HITS-CLIP	Functional MTI (Weak)	23824327
10799	Homo sapiens	HITS-CLIP	Functional MTI (Weak)	23824327
6241	Homo sapiens	qRT-PCR	Functional MTI (Weak)	24940696
90378	Homo sapiens	HITS-CLIP	Functional MTI (Weak)	23824327
389432	Homo sapiens	HITS-CLIP	Functional MTI (Weak)	23824327
25956	Homo sapiens	HITS-CLIP	Functional MTI (Weak)	19536157
57715	Homo sapiens	HITS-CLIP	Functional MTI (Weak)	23824327
10955	Homo sapiens	Luciferase repor	Functional MTI	24039954

462	Homo sapiens	HITS-CLIP	Functional MTI (Weak)	23824327
5345	Homo sapiens	HITS-CLIP	Functional MTI (Weak)	23824327
23544	Homo sapiens	PAR-CLIP	Functional MTI (Weak)	22012620
23544	Homo sapiens	PAR-CLIP	Functional MTI (Weak)	23446348
375035	Homo sapiens	HITS-CLIP	Functional MTI (Weak)	23824327
8879	Homo sapiens	PAR-CLIP	Functional MTI (Weak)	23592263
10019	Homo sapiens	HITS-CLIP	Functional MTI (Weak)	23824327
63898	Homo sapiens	HITS-CLIP	Functional MTI (Weak)	23824327
80851	Homo sapiens	HITS-CLIP	Functional MTI (Weak)	23824327
9644	Homo sapiens	HITS-CLIP	Functional MTI (Weak)	23824327
6474	Homo sapiens	HITS-CLIP	Functional MTI (Weak)	23824327
9121	Homo sapiens	HITS-CLIP	Functional MTI (Weak)	23824327
6505	Homo sapiens	HITS-CLIP	Functional MTI (Weak)	23824327
9356	Homo sapiens	HITS-CLIP	Functional MTI (Weak)	23824327
11001	Homo sapiens	HITS-CLIP	Functional MTI (Weak)	23824327
55238	Homo sapiens	HITS-CLIP	Functional MTI (Weak)	23824327
201266	Homo sapiens	HITS-CLIP	Functional MTI (Weak)	23824327
55334	Homo sapiens	HITS-CLIP	Functional MTI (Weak)	23824327
8501	Homo sapiens	HITS-CLIP	Functional MTI (Weak)	19536157
124935	Homo sapiens	HITS-CLIP	Functional MTI (Weak)	23824327
4091	Homo sapiens	HITS-CLIP	Functional MTI (Weak)	23824327
64093	Homo sapiens	HITS-CLIP	Functional MTI (Weak)	23824327
6591	Homo sapiens	HITS-CLIP	Functional MTI (Weak)	23824327
51429	Homo sapiens	HITS-CLIP	Functional MTI (Weak)	23824327
6648	Homo sapiens	PAR-CLIP	Functional MTI (Weak)	23446348
6648	Homo sapiens	PAR-CLIP	Functional MTI (Weak)	23592263
57537	Homo sapiens	PAR-CLIP	Functional MTI (Weak)	20371350
57537	Homo sapiens	PAR-CLIP	Functional MTI (Weak)	23592263
6655	Homo sapiens	HITS-CLIP	Functional MTI (Weak)	23824327
65124	Homo sapiens	HITS-CLIP	Functional MTI (Weak)	23824327
6667	Homo sapiens	HITS-CLIP	Functional MTI (Weak)	23824327
6689	Homo sapiens	HITS-CLIP	Functional MTI (Weak)	23824327
83985	Homo sapiens	HITS-CLIP	Functional MTI (Weak)	23824327
8405	Homo sapiens	HITS-CLIP	Functional MTI (Weak)	23824327
10847	Homo sapiens	HITS-CLIP	Functional MTI (Weak)	23824327
57522	Homo sapiens	HITS-CLIP	Functional MTI (Weak)	23824327
6749	Homo sapiens	Luciferase repor	Functional MTI	24039954
8614	Homo sapiens	HITS-CLIP	Functional MTI (Weak)	23824327
79689	Homo sapiens	HITS-CLIP	Functional MTI (Weak)	23824327
55342	Homo sapiens	HITS-CLIP	Functional MTI (Weak)	23824327
94056	Homo sapiens	HITS-CLIP	Functional MTI (Weak)	23824327
9145	Homo sapiens	HITS-CLIP	Functional MTI (Weak)	23824327
55333	Homo sapiens	HITS-CLIP	Functional MTI (Weak)	23824327
148281	Homo sapiens	HITS-CLIP	Functional MTI (Weak)	23824327
387332	Homo sapiens	PAR-CLIP	Functional MTI (Weak)	22012620
6938	Homo sapiens	Immunofluorescer	Functional MTI	22523078
6934	Homo sapiens	HITS-CLIP	Functional MTI (Weak)	23824327
9895	Homo sapiens	HITS-CLIP	Functional MTI (Weak)	23824327
7040	Homo sapiens	Luciferase repor	Functional MTI	23726841
7048	Homo sapiens	Luciferase repor	Functional MTI	24039954

9220	Homo sapiens	HITS-CLIP	Functional MTI (Weak)	23824327
11011	Homo sapiens	PAR-CLIP	Functional MTI (Weak)	22291592
79853	Homo sapiens	HITS-CLIP	Functional MTI (Weak)	23824327
23671	Homo sapiens	PAR-CLIP	Functional MTI (Weak)	22291592
80008	Homo sapiens	HITS-CLIP	Functional MTI (Weak)	23824327
653567	Homo sapiens	HITS-CLIP	Functional MTI (Weak)	23824327
160335	Homo sapiens	HITS-CLIP	Functional MTI (Weak)	23824327
26092	Homo sapiens	HITS-CLIP	Functional MTI (Weak)	23824327
11076	Homo sapiens	PAR-CLIP	Functional MTI (Weak)	20371350
11076	Homo sapiens	PAR-CLIP//HITS-(	Functional MTI (Weak)	21572407
11076	Homo sapiens	PAR-CLIP	Functional MTI (Weak)	23446348
11076	Homo sapiens	HITS-CLIP	Functional MTI (Weak)	23824327
9819	Homo sapiens	PAR-CLIP	Functional MTI (Weak)	22100165
55020	Homo sapiens	HITS-CLIP	Functional MTI (Weak)	23824327
158219	Homo sapiens	HITS-CLIP	Functional MTI (Weak)	23824327
10628	Homo sapiens	PAR-CLIP	Functional MTI (Weak)	22012620
51465	Homo sapiens	HITS-CLIP	Functional MTI (Weak)	23824327
7353	Homo sapiens	HITS-CLIP	Functional MTI (Weak)	23824327
54490	Homo sapiens	HITS-CLIP	Functional MTI (Weak)	23824327
201294	Homo sapiens	HITS-CLIP	Functional MTI (Weak)	23824327
22846	Homo sapiens	HITS-CLIP	Functional MTI (Weak)	23824327
7408	Homo sapiens	HITS-CLIP	Functional MTI (Weak)	23824327
10451	Homo sapiens	PAR-CLIP	Functional MTI (Weak)	20371350
10451	Homo sapiens	HITS-CLIP	Functional MTI (Weak)	21572407
10451	Homo sapiens	HITS-CLIP	Functional MTI (Weak)	23706177
10451	Homo sapiens	HITS-CLIP	Functional MTI (Weak)	24906430
57191	Homo sapiens	HITS-CLIP	Functional MTI (Weak)	23824327
10885	Homo sapiens	HITS-CLIP	Functional MTI (Weak)	23824327
8840	Homo sapiens	HITS-CLIP	Functional MTI (Weak)	23824327
80014	Homo sapiens	HITS-CLIP	Functional MTI (Weak)	23824327
55841	Homo sapiens	HITS-CLIP	Functional MTI (Weak)	23824327
114786	Homo sapiens	PAR-CLIP	Functional MTI (Weak)	20371350
143570	Homo sapiens	HITS-CLIP	Functional MTI (Weak)	23824327
26137	Homo sapiens	HITS-CLIP	Functional MTI (Weak)	23824327
9278	Homo sapiens	HITS-CLIP	Functional MTI (Weak)	23824327
23099	Homo sapiens	HITS-CLIP	Functional MTI (Weak)	19536157
728116	Homo sapiens	HITS-CLIP	Functional MTI (Weak)	19536157
219654	Homo sapiens	HITS-CLIP	Functional MTI (Weak)	19536157
253832	Homo sapiens	PAR-CLIP	Functional MTI (Weak)	20371350
253832	Homo sapiens	PAR-CLIP	Functional MTI (Weak)	21572407
253832	Homo sapiens	PAR-CLIP	Functional MTI (Weak)	23446348
253832	Homo sapiens	PAR-CLIP	Functional MTI (Weak)	23592263
253832	Homo sapiens	PAR-CLIP	Functional MTI (Weak)	24398324
463	Homo sapiens	PAR-CLIP	Functional MTI (Weak)	22012620
387032	Homo sapiens	HITS-CLIP	Functional MTI (Weak)	23824327
7570	Homo sapiens	HITS-CLIP	Functional MTI (Weak)	23824327
149076	Homo sapiens	HITS-CLIP	Functional MTI (Weak)	23824327
79750	Homo sapiens	HITS-CLIP	Functional MTI (Weak)	19536157
57541	Homo sapiens	HITS-CLIP	Functional MTI (Weak)	23824327
197407	Homo sapiens	HITS-CLIP	Functional MTI (Weak)	23824327

79177	Homo sapiens	PAR-CLIP	Functional MTI (Weak)	22291592
9831	Homo sapiens	HITS-CLIP	Functional MTI (Weak)	23824327
115509	Homo sapiens	HITS-CLIP	Functional MTI (Weak)	23824327
440077	Homo sapiens	HITS-CLIP	Functional MTI (Weak)	23824327
65988	Homo sapiens	HITS-CLIP	Functional MTI (Weak)	23824327
342945	Homo sapiens	HITS-CLIP	Functional MTI (Weak)	23824327
55055	Homo sapiens	HITS-CLIP	Functional MTI (Weak)	23824327
79699	Homo sapiens	PAR-CLIP	Functional MTI (Weak)	22012620

ated target number is 321.

es (PMID)
